# PPARG-centric transcriptional re-wiring during differentiation of human trophoblast stem cells into extravillous trophoblasts

**DOI:** 10.1093/nar/gkaf669

**Published:** 2025-07-24

**Authors:** Qingqing Guo, Joonhyuk Choi, Muyoung Lee, Jonghwan Kim

**Affiliations:** Department of Molecular Biosciences, The University of Texas at Austin, Austin, TX 78712, United States; Department of Molecular Biosciences, The University of Texas at Austin, Austin, TX 78712, United States; Department of Molecular Biosciences, The University of Texas at Austin, Austin, TX 78712, United States; Department of Molecular Biosciences, The University of Texas at Austin, Austin, TX 78712, United States; Center for Systems and Synthetic Biology, The University of Texas at Austin, Austin, TX 78712, United States

## Abstract

Peroxisome proliferator-activated receptor gamma (PPARG) is a nuclear receptor family transcription factor (TF) critical for adipogenesis, lipid metabolism, insulin sensitivity, and inflammation. It has also been known to play essential roles in trophoblast development and placentation. Dysregulation of PPARG in trophoblast differentiation has been implicated in pregnancy complications, such as pre-eclampsia and gestational diabetes. However, the molecular mechanisms of PPARG-dependent target gene regulation and its interactions with other regulatory factors during human trophoblast differentiation remain unclear. Using human trophoblast stem cells (TSCs), mimicking placental cytotrophoblasts (CTs), and their differentiation into extravillous trophoblasts (EVTs) as our models, we reveal that PPARG has cell-type-specific targets in TSCs and EVTs. We also find that while PPARG is essential for both TSC self-renewal and EVT differentiation, only its role in EVT differentiation is ligand sensitive and requires ligand-binding domain (LBD)-mediated transcriptional activity, whereas its function in TSC self-renewal appears to be ligand insensitive. Combined analysis with chromosomal targets of previously defined key TFs in TSCs and EVTs shows that PPARG forms trophoblast cell-type-specific regulatory circuitries, leading to differential target gene regulation via transcriptional re-wiring during EVT differentiation. Additionally, the enhanced invasiveness of EVTs treated with a PPARG agonist suggests a potential connection between PPARG pathways and human placenta accreta.

## Graphical Abstract

**Graphical Abstract:**
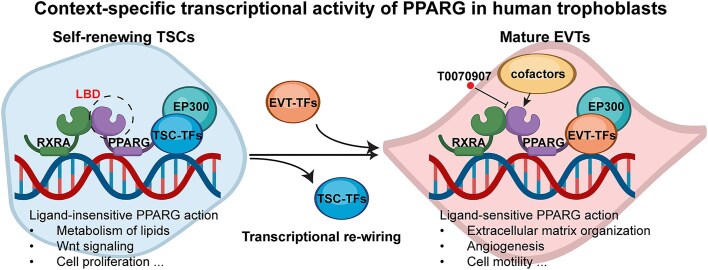

## Introduction

The placenta is a multifunctional organ that plays a crucial role during pregnancy by connecting the developing fetus to the maternal tissue [1, 2]. It consists of multiple trophoblast cell types originating from the trophectoderm of the developing embryo [3, 4]. The human placental trophoblasts include cytotrophoblasts (CTs), which are stem cell/progenitor populations, as well as more specialized cell types such as syncytiotrophoblasts (STs) and extravillous trophoblasts (EVTs) [3, 5]. Defects in trophoblast differentiation can lead to early pregnancy failure or various pregnancy-related disorders, impacting both pregnancy and postnatal health, and resulting in significant socioeconomic burdens [4, 6, 7]. Understanding normal and abnormal human placental development is crucial for improving our knowledge and treating placenta-associated complications. However, studying human placental development *in vivo* has been limited due to ethical, legal, and technical issues. Recent advances, including the establishment of multiple *in vitro* models, such as human trophoblast stem cells (TSCs) and *trans*-differentiated human trophoblast stem-like cells (TSLCs) from pluripotent stem cells, now provide powerful *in vitro* tools [5, 8, 9]. With their confirmed bipotency to differentiate into STs and EVTs *in vitro*, these models allow for detailed molecular-level characterization of human trophoblast lineage development and placentation.

Peroxisome proliferator-activated receptor gamma (PPARG) plays crucial roles in various cellular processes, including lipid and glucose metabolism and inflammation [10, 11]. PPARG belongs to the nuclear receptor family that can function in both ligand-dependent and ligand-independent manners, depending on the cell type and context [12, 13]. Ligand-dependent activation of PPARG is mediated through its ligand-binding domain (LBD), which facilitates conformational changes required for coactivator recruitment and transcriptional regulation [12, 13]. Endogenous ligands, such as fatty acids and their derivatives, play a crucial role in modulating its activity [14, 15]. While PPARG’s role has been extensively investigated in other cell types, such as adipocytes and macrophages, its specific roles in regulating trophoblast self-renewal and differentiation during human placental development require further investigation. In the context of trophoblasts, studies using various animal and previous *in vitro* models have suggested that PPARG significantly affects trophoblast differentiation, placenta development, endometrium decidualization, and implantation [16–18]. PPARG has also been implicated in pregnancy-related complications, such as pre-eclampsia (PE), intrauterine growth restriction, and other placental pathologies [16–18]. However, prior reports have shown discrepancies. For example, some studies reported that PPARG hinders EVT differentiation by forming a heterodimer with its interacting partner retinoid X receptor alpha (RXRA) [16, 19], whereas other studies indicated that PPARG promotes trophoblast invasion in the HTR8/SVneo trophoblast cell line [20, 21]. In mouse TSC studies, the PPARG agonist Rosiglitazone (Rosi) shifted differentiation to the labyrinthine lineage while inhibiting trophoblast invasion [22, 23]. Conversely, other studies suggested that Rosi could treat PE, a condition characterized by shallow invasion caused by defective EVT differentiation with decreased PPARG expression [24, 25]. The inconsistencies are likely to be due to the use of different species, *in vivo* versus *in vitro* models, different trophoblast cell types, and varying environmental contexts. This underscores the need for further research to clarify the precise roles of PPARG and its regulatory mechanisms across multiple trophoblast cell types, in particular to achieve a comprehensive understanding of the regulatory mechanisms of human PPARG.

In this current study, we utilized recently established human TSCs, which robustly mimic human placental trophoblasts, to explore the critical role of PPARG in maintaining the stemness of TSCs and EVT differentiation [5]. We first confirmed that PPARG is essential for both self-renewal of TSCs and EVT differentiation. Interestingly, while its role in TSC self-renewal appears ligand insensitive, PPARG function during EVT differentiation becomes ligand sensitive and requires LBD-mediated transcriptional activity. Our results additionally suggested that PPARG collaborates with recently reported TSC or EVT-specific key transcription factors (TFs) to control trophoblast cell-type-specific target genes. The results strongly indicate that PPARG forms distinct regulatory circuitries for trophoblast cell-type-specific functions. Addressing prior discrepancies, we demonstrate that the PPARG agonist enhances the invasiveness of human EVTs. All these findings suggest that PPARG plays a pivotal role during human trophoblast specification, clarifying its involvement in controlling trophoblast cell functions.

## Materials and methods

### Cell culture

The TSC line CT27, derived from the first-trimester human placenta, was kindly provided by Hiroaki Okae (Department of Informative Genetics Environment and Genome Research Center, Tohoku University Graduate School of Medicine, Japan). The cells were maintained and differentiated as previously described [5]. For EVT differentiation, cells were cultured on plates coated with 1 μg/ml Collagen IV (Corning, 354233), achieving mature EVT differentiation by day 8. For ST (3D) differentiation, cells were cultured in a 6 cm Petri dish, with mature differentiation reached by day 6.

### Generation and differentiation of trophoblast organoids

The generation of trophoblast organoids was performed based on established protocols [26]. Briefly, TSCs were seeded into aggrewell 800 (STEMCELL Technologies, 34815) in 2 ml of pre-culture medium. On day 2, the medium was replaced with a weak differentiation medium and refreshed on day 4. On day 5, the medium was replaced with a strong differentiation medium and refreshed on day 7. On day 8, the organoids were collected and resuspended in Matrigel (Corning, 354234) for further differentiation. The differentiation was conducted using conditions previously described [5]. After 8 days of organoid generation (day 8 + 0), the organoids were collected and seeded into a 24-well culture plate in 30 μl of Matrigel droplets per well. The organoids were differentiated in EVT differentiation medium, achieving maturity by day 8 + 8 to 9.

### Drug treatment

Drugs were added at the indicated times and maintained as described. Compounds were dissolved in dimethylsulfoxide (DMSO; Sigma-Aldrich, D8418) or H_2_O, as appropriate. The following were used: T0070907 (Selleck Chemicals, S2871; 1 μM), Rosiglitazone (MedChemExpress, HY-17386; 1 μM), LGD1069 (Selleck Chemicals, S2098; 1 μM), CD3254 (Fisher Scientific, 33-025-0; 1 μM), Troglitazone (MedChemExpress, HY-50935; 1 μM), DMOG (Selleck Chemicals, S7483; 500 μM), and SCH772984 (MedChemExpress, HY-50846; 0.5 μM).

### shRNA knockdown

Short hairpin RNAs (shRNAs) targeting PPARG and RXRA, as well as non-specific shRNAs, were acquired from Sigma-Aldrich. HEK293T cells were cultured in a 12-well plate in Dulbecco’s modified Eagle’s medium (DMEM; Thermo Fisher Scientific, 11965118) with 10% fetal bovine serum (FBS; Sigma-Aldrich, 12306C) and 0.5% penicillin–streptomycin (Thermo Fisher Scientific, 15140-122). After reaching 50–70% confluency, cells were transfected with 375 ng of the pLKO construct containing specific shRNA, 250 ng of the Δ8.9 plasmid, and 125 ng of the VSVG plasmid using 2.25 μl of GeneJet transfection reagent (SignaGen Laboratories, SL100488). Lentiviruses were harvested 24 and 48 h after transfection and purified by centrifugation at 800 rpm for 3 min. For knockdown (KD) in TSCs, cells at 30–50% confluency were infected with lentiviral solutions with the addition of polybrene (Santa Cruz Biotechnology, sc-134220) at a final concentration of 5 μg/ml. At 18 h post-infection, cells were cultured in TSC medium supplemented with 1 μg/ml puromycin (Thermo Fisher Scientific, A1113803) for drug selection. For KD during EVT lineage, cells were infected with lentiviral solutions on EVT day 0 or day 1. Medium containing 1 μg/ml puromycin was replaced 18 h post-infection. The shRNA sequences are available in Supplementary Table S1.

### Colony formation

TSCs were infected with PPARG-targeting shRNAs (KD1 or KD2) or a non-targeting control, followed by puromycin selection as described. Cells were then seeded at low density (4000 cells per well) in 24-well plates and maintained under standard TSC conditions for 3 days without passaging. Colonies were fixed with 100% methanol at room temperature for 15 min and stained with 0.5% crystal violet (Sigma-Aldrich, C0775) for another 15 min. After rinsing with distilled water to remove excess dye, plates were air-dried and imaged using the Typhoon™ FLA 9500 scanner. Colony area was quantified in ImageJ by applying a consistent threshold to grayscale images to calculate the percentage coverage per well. Three biological replicates were analyzed for each condition.

### Cell proliferation (CCK-8)

After lentiviral infection and puromycin selection as described above, TSCs were seeded at 2500 cells per well into 96-well plates. Cell proliferation was measured daily for four consecutive days using the Cell Counting Kit-8 (Dojindo, CK04), according to the manufacturer's instructions. At each time point, 10 μl of CCK-8 reagent was added to each well and incubated for 2 h at 37°C. Absorbance at 450 nm was measured using a Tecan M1000 plate reader. Three biological replicates were analyzed for each condition.

### Quantitative real-time PCR (RT-qPCR)

Total RNA was extracted from cells using the RNeasy Plus Mini Kit (250) (Qiagen, 74136). For each sample, 500 ng of RNA was reverse transcribed into cDNA with qScript™ cDNA SuperMix (Quantabio, 95048-100). The cDNA was diluted to a 1:20 ratio, and RT-qPCR was performed using PowerTrack™ SYBR Green Master Mix (Thermo Fisher Scientific, A46112) in a StepOnePlus Real-Time PCR System (Applied Biosystems). Relative expression was calculated using the ΔΔCt method, normalized to the housekeeping gene glyceraldehyde phosphate dehydrogenase (GAPDH). *P*-values were determined using three biological replicates via Student's *t*-test, with significance levels annotated: *, **, and *** for *P*-value < 0.05, *P*-value < 0.01, and *P*-value < 0.001, respectively. The primers used are listed in Supplementary Table S1.

### Western blotting

Proteins were extracted and denatured using Laemmli Sample Buffer (Bio-Rad, 1610747) by heating at 98°C for 10 min. After electrophoresis on a 10% sodium dodecylsulfate (SDS)–polyacrylamide gel electrophoresis (PAGE) gel, proteins were transferred onto a polyvinylidene fluoride (PVDF) membrane (MilliporeSigma, IPVH00010). The membrane was then blocked with 5% skim milk (Bio-Rad, 1706404) in TBS-T (Tris-buffered saline with 0.1% Tween-20) for 2 h and incubated overnight at 4°C with the primary antibody. After three washes in TBS-T, the membrane was incubated with a horseradish peroxidase (HRP)-conjugated secondary antibody for 1 h at room temperature. The membrane was washed three more times in TBS-T, and protein detection was performed using ECL Western blotting reagent (Thermo Fisher Scientific, 45-002-401) and visualized with ChemiDoc XRS+ (Bio-Rad). The primary antibodies used for western blotting were as follows: PPARG (Cell Signaling Technology, 2443S; 1:1000), RXRA (Cell Signaling Technology, 3085S; 1:1000), EP300 (Abcam, ab10485; 1:1000), MED1 (Novus Biologicals, NB100-2574; 1:1000), MED15 (Proteintech, 11566-1-AP; 1:1000), MSX2 (Novus Biologicals, NBP1-85445; 1:1000), DLX5 (Abcam, ab109737; 1:1000), HIF1A (Cell Signaling Technology, 36169S; 1:1000), GCM1 (Abcam, ab187860; 1:1000), and ACTB (Abcepta, AM1829B; 1:2000). The secondary antibodies were HRP-linked anti-rabbit IgG (Cell Signaling Technology, 7074S; 1:10 000) and HRP-linked anti-mouse IgG (Cell Signaling Technology, 7076S; 1:10 000).

### Co-immunoprecipitation followed by western blot

TSCs were cultured under standard conditions and harvested at ∼80% confluency. For each co-immunoprecipitation (co-IP) experiment, two 15 cm dishes were used: one for PPARG immunoprecipitation and one as a control. Cells were washed with cold phosphate-buffered saline (PBS) and lysed in ice-cold Pierce™ IP Lysis Buffer (Thermo Fisher Scientific, 87 788) with protease inhibitor cocktail (Sigma-Aldrich, 11836170001) for 1 h at 4°C on a rotator. Lysates were cleared by centrifugation at 13,000 rpm for 15 min at 4°C. For immunoprecipitation, the PPARG antibody (Cell Signaling Technology, 2443S, 1:50) was conjugated to Dynabeads™ Protein A (Thermo Fisher Scientific, 10001D) according to the manufacturer's instructions. Beads incubated without antibody were used as a control to assess non-specific binding. Cleared lysates were incubated with antibody-conjugated beads overnight at 4°C with gentle rotation. The next day, beads were washed three times with Pierce™ IP Lysis Buffer, twice with TBS, and then resuspended in 2× Laemmli Sample Buffer (Bio-Rad, 1610747) and boiled at 98°C for 10 min. For western blotting analysis, 1% of the pre-IP lysate (10 μl out of 1 ml total) was saved as the input sample. Western blot was performed as previously described.

### Matrigel-based invasion assay

The invasion assay was conducted according to a previously established protocol with slight modifications [27]. Secure Seal Hybridization Chambers (Thermo Fisher Scientific, S24732) were sterilized under UV light for 1 h and then adhered to the bottom of a 12-well plate. A 2:1 mixture of Matrigel and EVT differentiation medium was added to the chambers and equilibrated in a 37°C incubator for 1 h. Then 5 × 10^4^ cells suspended in 30 μl of EVT differentiation medium were introduced to each chamber and attached to the Matrigel surface. The plate was positioned vertically in the incubator for 1 h, after which 1 ml of EVT differentiation medium was added to each well. The EVT invasion ability was assessed on day 7 or day 8.

### RNA-seq

mRNAs were isolated using the NEBNext® Poly(A) mRNA Magnetic Isolation Module (New England Biolabs, E7490L), and cDNA libraries were prepared with the NEBNext® Ultra™ RNA Library Prep Kit for Illumina (New England Biolabs, E7770) following the manufacturer's instructions. The RNA-seq libraries were sent to Novogene for paired-end sequencing with 150 bp read length on an Illumina NovaSeq 6000. Published RNA-seq data were downloaded from the Gene Expression Omnibus (GEO) (GSE212266). All sequencing reads were trimmed using Trim Galore (https://github.com/FelixKrueger/TrimGalore) (version 0.6.10) and aligned to the human reference genome (hg38) with STAR (version 2.7.11b) [28]. Alignments were then filtered using Samtools (version 1.17) with MAPQ score ≥ 10 [29]. Raw counts were quantified using featureCounts from the Subread package (version 2.0.6) [30] and normalized with the DESeq2 R package (version 1.30.1) [31]. The differentially expressed genes (DEGs) were identified with adjusted *P*-value < 0.05 and absolute log2 fold change (FC) > 1. Gene expression levels were also quantified in transcripts per million (TPM) using Salmon (version 0.13.1) [32] and the R package tximport (version 1.18.0) [33].

### ChIP-seq

Cells were fixed with 1% formaldehyde for 7 min at room temperature, followed by the addition of glycine to a final concentration of 125 mM to quench the reaction. Genomic DNA was sonicated to 200 bp using a Bioruptor (Diagenode), and cell lysates were pre-cleaned with 50 μl of Protein A agarose (Sigma-Aldrich, 11134515001). Subsequently, 10 μg of primary antibody was added to each sample, which was nutated at 4°C overnight for immunoprecipitation. The next day, 60 μl of Protein A agarose was added to each sample for the pull-down of target proteins. The enriched DNAs were then used to generate sequencing libraries with the NEBNext® Ultra™ DNA Library Prep Kit (New England Biolabs, E7645S) and sequenced in paired-end mode with 150 bp read length on an Illumina NovaSeq 6000. The antibodies used for chromatin immunoprecipitation sequencing (ChIP-seq) were PPARG (Cell Signaling Technology, 2443S, 10 μl), RXRA (Cell Signaling Technology, 3085S, 10 μl), EP300 (Abcam, ab10485, 10 μl), NCOA3 (Cell Signaling Technology, 2126S, 10 μl), MED1 (Novus Biologicals, NB100-2574, 10 μl), MED12 (Bethyl Laboratories, A300-774A, 10 μl), MED15 (Proteintech, 11566-1-AP, 10 μl), and H3K27ac (Cell Signaling Technology, 8173S, 10 μl).

Published ChIP-seq and ATAC-seq (assay for transposase-accessible chromatin sequencing) data were downloaded from the GEO (GSE64458; GSE208539; GSE212265). All data reads were trimmed with Trim Galore (version 0.6.10) and mapped to the hg38 human reference genome using Bowtie2 (version 2.5.1) with default parameters [34]. The alignments were further filtered with Samtools (version 1.17) with MAPQ ≥ 10 [29]. PCR duplicates were subsequently removed with Picard Tools (version 2.27.2) (https://broadinstitute.github.io/picard/). Peaks were detected utilizing MACS3 (version 3.0.0b3) [35] with a false discovery rate (FDR) < 0.05, and peaks were annotated using HOMER (version 4.11) (http://homer.ucsd.edu/homer/). Normalized ChIP-seq read counts were calculated using HOMER’s annotatePeaks.pl (±1 kb from PPARG summits) with tag directories generated from two biological replicates. Mean signal values from the two replicates were used for plotting and statistical comparisons. For visualization, RPGC-normalized bigwig files were produced using the deepTools bamCoverage (version 3.5.2) [36]. The differential binding analysis was performed using DiffBind (version 3.14.0) [37] with adjusted *P*-value < 0.05 and absolute log2 FC > 1.

### Immunofluorescence

Cells were fixed with 4% paraformaldehyde in cold D-PBS (PBS containing Ca^2+^ and Mg^2+^) for 20 min. After three washes with D-PBS/0.1% bovine serum albumin (BSA), the cells were blocked by 10% horse serum in PBS/0.1% BSA with 0.3% Triton X-100 (Sigma-Aldrich, T8787) for 45 min at room temperature. The cells were then incubated overnight at 4°C with the primary antibody in D-PBS/0.1% BSA with 10% horse serum, followed by three washes with D-PBS/0.1% BSA. Subsequently, cells were incubated with Alexa Fluor 594-conjugated secondary antibodies (Invitrogen, A-11037) for 2 h at room temperature. After three washes to remove excess antibodies, the nuclei were stained with 4′,6-diamidino-2-phenylindole (DAPI; Sigma-Aldrich). Confocal microscopy images were captured using a Nikon W1 Spinning Disk Confocal Microscope. All samples within the same experiment were imaged under consistent immunofluorescence (IF) microscopy settings. The primary antibody used for IF was PPARG (Cell Signaling Technology, 2443S, 1:200).

## Results

### Trophoblast cell-type-specific expression patterns of PPARG *in vivo* and *in vitro*

**Figure 1.**
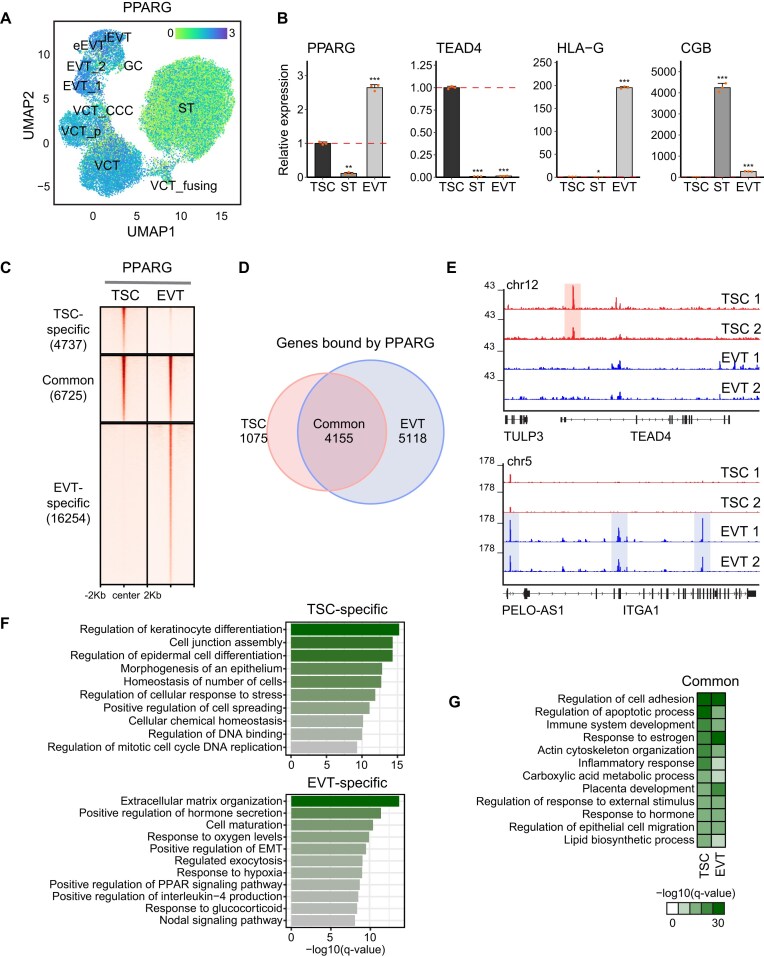
Cell-type-specific expression and binding patterns of PPARG during trophoblast development. (**A**) UMAP of snRNA-seq data from first-trimester human placenta showing the expression patterns of PPARG along the EVT and ST lineages. (**B**) Quantitative real-time PCR (RT-qPCR) analysis displaying the expression levels of TSC (TEAD4), EVT (HLA-G), and ST (CGB3) marker genes, and PPARG in self-renewing TSCs and differentiated EVTs and STs. *P*-values were calculated using Student's *t*-test with three biological replicates; *, **, and *** indicate *P*-value < 0.05, 0.01, and 0.001, respectively. (**C**) Total PPARG-binding loci were clustered into three groups: TSC-specific, common, and EVT-specific, based on the results of differential binding analysis using DiffBind (cut-off: |log2FC| > 1, adjusted *P*-value < 0.05, Wald test with Benjamini–Hochberg correction). Heatmap showing the ChIP-seq signals of PPARG in TSCs and EVTs across the clustered loci. (**D**) Venn diagram of the genes targeted by PPARG in either TSCs or EVTs. (**E**) Gene track view displaying PPARG ChIP-seq signals in TSCs and EVTs near the TSC marker (TEAD4) and EVT marker (ITGA1). (**F**) Gene Ontology (GO) enrichment analysis of PPARG-binding sites specific to TSCs or EVTs. (**G**) GO enrichment analysis of PPARG-binding sites shared between TSCs and EVTs, conducted using the GREAT program.

To confirm the specific human trophoblast cell types where PPARG is predominantly expressed, we first analyzed publicly available single-nuclei RNA-seq (snRNA-seq) data obtained from an early trophoblast donor at the maternal–fetal interface [38]. The UMAP and violin plots revealed a clear lineage progression from CT (VCT) to either the EVT or ST lineage, evidenced by the elevated expression of lineage-specific markers [human leukocyte antigen-G (HLA-G) for EVT and CGB3 for ST] (Supplementary Fig. S1A, B). The analysis also confirmed that PPARG is expressed in placental CTs (with highly expressed TP63) and maintains even higher expression levels in EVT cell types (Fig. 1A; Supplementary Fig. S1A). Notably, while the importance of PPARG has been implicated in the ST lineage [39–41], the expression level of PPARG is significantly reduced in placental STs (Fig. 1A; Supplementary Fig. S1A). To assess whether the *in vivo* expression patterns of PPARG in human placental cell types match the expression profiles observed in *in vitro* model systems, we tested the expression levels of PPARG in self-renewing TSCs and during ST and EVT differentiation. The successful differentiation into these cell types was confirmed by measuring the expression of the EVT-specific marker HLA-G and the ST-specific marker CGB3 (Fig. 1B). Additional western blot results revealed that PPARG is highly expressed in self-renewing TSCs, with even higher expression in EVTs (Supplementary Fig. S1C). In contrast, a significant reduction in PPARG expression was observed in STs, just as observed *in vivo* (Fig. 1B; Supplementary Fig. S1C). In both TSCs and EVTs, PPARG is mainly located in the nuclei (Supplementary Fig. S1E). To further explore the dynamic changes in PPARG expression during ST and EVT differentiation, we analyzed time-course RNA-seq data [42], revealing a gradual increase in PPARG levels transitioning from TSCs to EVTs, while an apparent down-regulation was observed during ST differentiation (Supplementary Fig. S1D). All these results revealed the expression patterns of PPARG in human trophoblast cell types. Additionally, *in vitro* expression patterns of PPARG are consistent with the expression patterns noted in our *in vivo* analysis, further confirming that *in vitro* differentiation of human TSCs robustly recapitulates *in vivo* trophoblast differentiation.

### Cell-type-specific target occupancy patterns of PPARG in TSCs and mature EVTs

Given the consistent expression patterns of PPARG in *in vivo* and *in vitro* models of placental cell types and noting that PPARG levels are relatively low in STs, we decided to focus on the roles of PPARG in TSCs and EVTs in the current study. With PPARG’s suggested versatile roles, we hypothesized that PPARG plays cell-type-specific roles via controlling cell-type-specific target genes. Notably, while transcriptional targets of PPARG have been reported in multiple cellular contexts [43, 44], its direct chromosomal targets in trophoblast cell types, such as TSCs and EVTs, have not been identified, especially in the human context. We conducted ChIP-seq using a native PPARG antibody in TSCs and EVTs. In accordance with its expression patterns (Fig. 1A, B; Supplementary Fig. S1A–D), PPARG occupies many more target loci in EVTs than in TSCs (11,462 and 22,979 loci in TSCs and EVTs, respectively) (Supplementary Table S2). In both cell types, PPARG exhibits a binding preference for distal regions, which is a typical target occupancy pattern of cell-type-specific TFs (Supplementary Fig. S1F). We also observed that while PPARG has many common targets between TSCs and EVTs, it also shows cell-type-specific occupancy patterns (Fig. 1C, D). As examples, PPARG occupies *cis-*regulatory elements of the TSC marker, TEAD4, in TSCs but not in EVTs. Conversely, *cis-*regulatory elements of the EVT marker ITGA1 are occupied by PPARG only in EVTs (Fig. 1E). Gene Ontology (GO) analysis of the genes associated with TSC-specific, EVT-specific, and common target loci (Fig. 1C) revealed the enrichment of distinct terms. Notably, keratinocyte differentiation and epidermal cell differentiation were enriched in TSC-specific loci-associated genes (Fig. 1F). Many terms implicated with known EVT functions, such as extracellular matrix, cell maturation, response to oxygen, epithelial to mesenchymal transition (EMT), and PPAR and nodal pathways were enriched in EVT-specific loci-associated genes (Fig. 1F). The genes associated with common target loci were enriched in previously known roles of PPARG, such as immune system development, inflammatory response, lipid biosynthetic processes, and placental development (Fig. 1G). Additionally, motif analysis of the target loci of PPARG in TSCs and EVTs using the HOMER program [45] found that, besides the PPAR::RXR motif, there was an enrichment of GATA, TEAD, and AP2 motifs in both cell types, with AP1, CEBP, and GCM motifs specifically enriched in EVTs (Supplementary Fig. S2A) [45]. This suggests that PPARG may form cooperative regulatory networks with other important trophoblast-specific TFs responsible for the identity of human trophoblast cell types [46, 47]. To further confirm cell-type-specific roles of PPARG, we additionally compared the targets of PPARG in TSCs with those in adipocytes [48]. As anticipated, we found cell-type-specific targets as well as common targets of PPARG (Supplementary Fig. S2B). The target occupancy of PPARG in adipocytes is greatly different from those in TSCs and EVTs (Supplementary Fig. S2C). The GO term analysis also confirmed that the top PPARG-bound regions in TSCs and adipocytes are enriched in distinct terms (Supplementary Fig. S2D), implying cell-type-specific roles of PPARG. Since distal regulatory elements or enhancers interact with cell-type-specific TFs to form enhanceosomes [49–51], our results suggest that PPARG’s regulatory roles are highly context-dependent and vary significantly across different cell types, even among human trophoblast cell types such as TSCs and EVTs.

### PPARG regulates TSC self-renewal through ligand-insensitive transcriptional mechanisms

**Figure 2.**
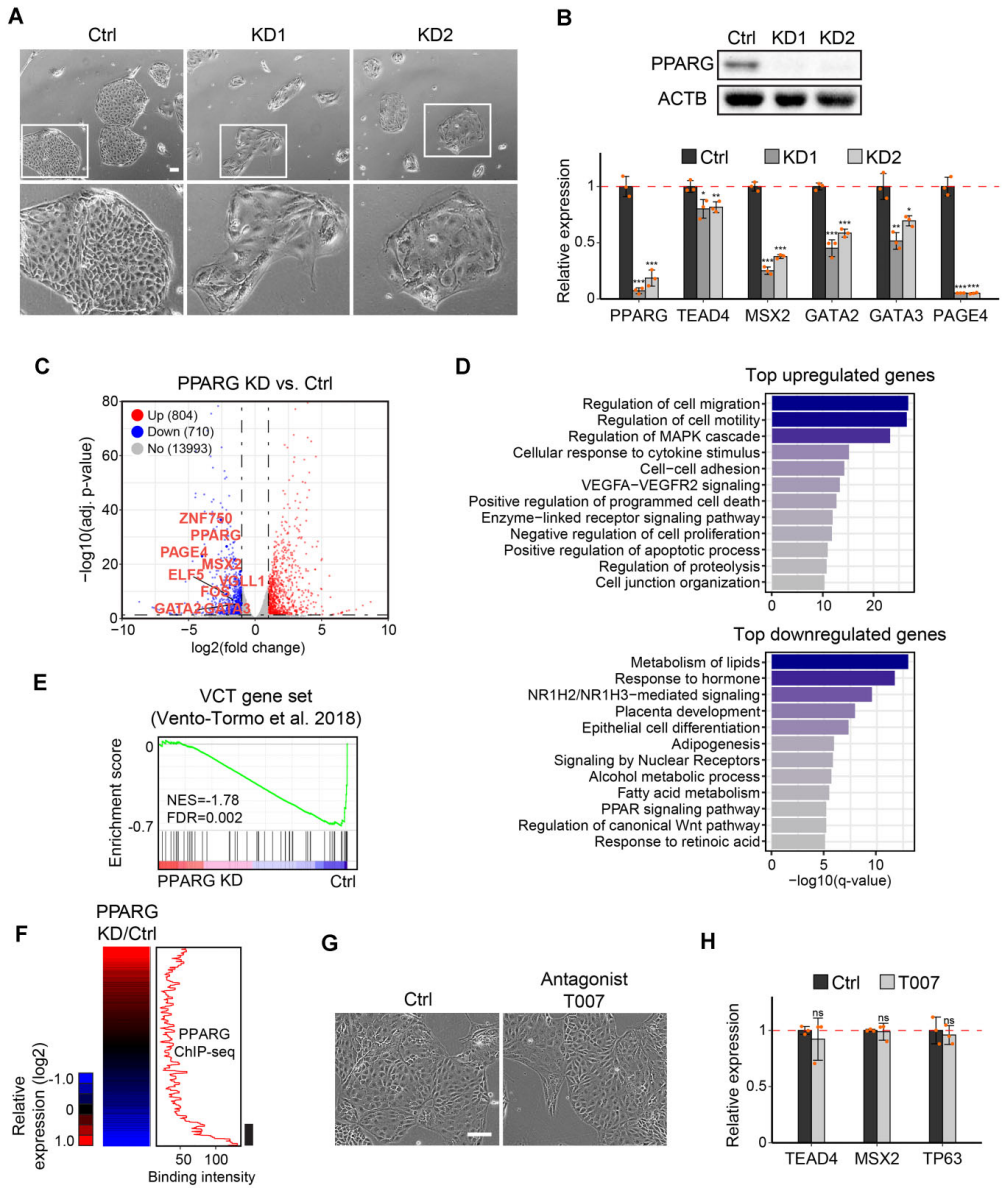
PPARG depletion, rather than antagonist treatment, disrupts TSC self-renewal. (**A**) Brightfield images showing the TSC colonial morphology under control and PPARG KD conditions (scale bar = 100 μm). (**B**) Western blot analysis showing successful depletion of PPARG in TSCs, and RT-qPCR results showing the relative expression of PPARG and TSC marker genes in PPARG KD cells compared with control TSCs. (**C**) Volcano plot depicting differentially expressed genes (DEGs) between PPARG KD and control TSCs 4 days post-lentivirus infection (cut-off: |log2FC| > 1, adjusted *P*-value < 0.05, Wald test with Benjamini–Hochberg correction). (**D**) GO enrichment analysis of the top down-regulated and up-regulated genes in PPARG KD compared with control TSCs. (**E**) Gene set enrichment analysis (GSEA) of PPARG KD compared with the control group using the CT marker gene set identified from scRNA-seq data of human first-trimester placenta. (**F**) Heatmap showing the log2-normalized expression of genes upon PPARG KD (left). Genes were ordered based on their relative expression levels in PPARG KD versus control. The PPARG occupancy signal for the ordered genes was plotted as the moving window average (window size, 100; bin size, 1) (right). (**G**) Brightfield images of cells treated with T007 for 72 h compared with control TSCs (scale bar = 100 μm). (**H**) Relative expression of TSC marker genes in T007-treated cells compared with control. *P*-values were calculated using Student's *t*-test with three biological replicates; *, **, and *** indicate *P*-value < 0.05, 0.01, and 0.001, respectively; ns, not significant.

As PPARG is expressed in both TSCs and EVTs, we first sought to evaluate the role of PPARG in self-renewing TSCs via shRNA-mediated KD using two different shRNAs. Compared with control TSCs which form tightly clustered epithelial colonies, TSCs upon PPARG KD displayed abnormal morphology and impeded colony growth (Fig. 2A). Western blot and RT-qPCR revealed successful depletion of PPARG by ∼80% for both shRNAs, and the KD of PPARG led to decreased expression of previously identified TSC marker genes, such as TEAD4, MSX2, GATA2, and TFAP2C [52, 53], indicating the loss of TSC identity (Fig. 2B). In addition, PPARG KD led to a significant reduction in colony formation and cell proliferation, as evidenced by colony formation and CCK-8 assays (Supplementary Fig. S2E, F).

To attain deeper insights into the expression profile caused by PPARG depletion, we performed RNA-seq for TSCs upon PPARG KD as well as for control cells. By differential expression analysis using DESeq2 [31], we identified 804 up- and 710 down-regulated genes in PPARG KD cells versus control (cut-off: |log2FC| > 1, adjusted *P*-value < 0.05) (Fig. 2C; Supplementary Table S3). Among the significantly down-regulated genes, we noted multiple genes that were previously implicated in TSC or CT functions, such as MSX2, GATA2, FOS, and VGLL1 [52–54] (Fig. 2C). The GO term analysis showed that upon PPARG KD, genes related to lipid and fatty acid metabolism are down-regulated, which is in line with the well-known function of PPARG [14]. Genes involved in placental development and those associated with WNT signaling were also enriched within the down-regulated gene set (Fig. 2D). On the other hand, the genes up-regulated by PPARG depletion were associated with cell migration, motility, and apoptosis, indicating the loss of self-renewal (Fig. 2D). To further verify the defective self-renewal, we performed a gene set enrichment analysis (GSEA) using a publicly available VCT-specific gene set from scRNA-seq of early human placenta [55] and observed that these genes predominantly exhibit a trend toward down-regulation upon PPARG KD (Fig. 2E), further supporting that PPARG is indispensable for maintaining stemness of human TSCs. Furthermore, we evaluated the expression levels of previously defined TSC-active TFs associated with super-enhancers (SEs) [53] in PPARG KD cells, and found that many such TFs are down-regulated upon PPARG KD, suggesting that PPARG is important for proper TSC self-renewal (Supplementary Fig. S2G). Since it is unclear whether PPARG functions as an activator or repressor to maintain TSC self-renewal, we performed a combined analysis of gene expression profiles upon KD and target occupancy in TSCs. The results revealed that PPARG preferentially occupies the genes that are down-regulated upon its depletion, indicating that PPARG acts as an activator for TSC self-renewal (Fig. 2F).

To further validate the specificity of PPARG KD, we generated a doxycycline (Dox)-inducible SBFB cell line expressing an shRNA-resistant PPARG construct by introducing mutations into the shRNA target region [56]. A FLAG-Bio tag was used to distinguish exogenous from endogenous PPARG. Upon Dox induction, western blot confirmed efficient KD of endogenous PPARG (lower band) with stable expression of the exogenous construct (upper band) (Supplementary Fig. S3A). Re-expression of PPARG partially rescued the self-renewal defects caused by KD, as evidenced by improved colony morphology and increased cell proliferation (Supplementary Fig. S3B, C). Expression of TSC marker genes showed partial recovery, and additional GSEA revealed enrichment of VCT marker genes and SE-TFs in the rescue group (Supplementary Fig. S3D, E). This supports that the observed KD phenotypes are due to the specific loss of PPARG rather than to off-target effects.

Prior studies have suggested that PPARG can activate target gene transcription through mechanisms that may involve or bypass LBD-mediated transcriptional regulation [44, 57]. To investigate how PPARG controls TSC self-renewal, we treated the cells with the selective PPARG antagonist T0070907 (T007) [58], which binds to the LBD, prevents ligand-induced conformational changes, and selectively inhibits LBD-mediated transcriptional activation. Interestingly, T007 treatment (1 μM) had no significant effect on TSC colony formation or the expression of self-renewal markers such as TEAD4, MSX2, and TP63 (Fig. 2G, H). Potential concentration-dependent effects were evaluated through dose–response experiments, which showed that TSC colony formation remained largely unaffected across a broad concentration range (0.3–10 μM) (Supplementary Fig. S3F). However, at 10 μM, we observed a modest reduction in proliferation, probably due to non-specific cytotoxicity rather than targeted inhibition of PPARG, as previously reported in other cell types (e.g. adipocytes and cancer cells) [59]. At 30 μM, TSCs experienced widespread cell death, further reinforcing the presence of off-target toxicity at high doses (Supplementary Fig. S3F). Transcriptome analysis also confirmed that PPARG KD samples clustered separately from both control and T007-treated TSCs (Supplementary Fig. S3G). The lack of a significant response to T007 suggests that PPARG’s role in maintaining TSC self-renewal may be largely ligand insensitive and not strictly dependent on LBD-mediated transcriptional activation.

### Depletion of PPARG results in defective EVT differentiation

**Figure 3.**
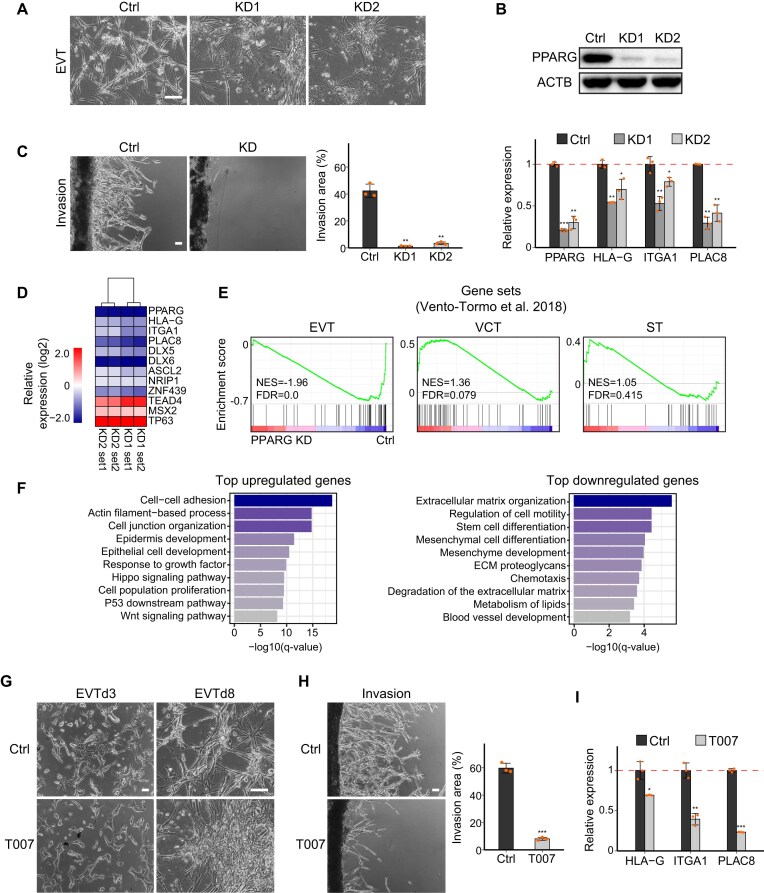
Impairment of EVT differentiation by PPARG depletion or antagonist treatment. (**A**) Brightfield images showing the EVT morphology under control and PPARG KD conditions, on EVT day 8 (scale bar = 100 μm). (**B**) Western blot analysis showing the reduced PPARG expression by shRNA-mediated KD on EVT day 8. RT-qPCR results showing the relative expression of PPARG and EVT marker genes in PPARG KD cells compared with control on EVT day 8. (**C**) Invasion assay of EVTs under control and PPARG KD conditions on day 8 (scale bar = 100 μm). (**D**) Heatmap of log2-normalized TPM expression of TSC and EVT marker genes in PPARG KD EVTs. Expression values were normalized to the mean of control EVTs. RNA-seq was performed on day 8 of EVT differentiation, corresponding to the terminal stage. (**E**) GSEA of PPARG KD compared with the control group, using the CT, EVT, and ST marker gene sets identified from scRNA-seq data of human first-trimester placenta. (**F**) GO enrichment analysis of the top down-regulated and up-regulated genes upon PPARG KD. (**G**) Brightfield images showing control and T007-treated cells on EVT day 3 (EVTd3) and day 8 (EVTd8) (scale bar = 100 μm). (**H**) Invasion assay of control and T007-treated EVTs on day 8 (scale bar = 100 μm). (**I**) Relative expression levels of EVT marker genes in T007-treated cells compared with control EVTs. *P*-values were calculated using Student's *t*-test with three biological replicates; *, **, and *** indicate *P*-value < 0.05, 0.01, and 0.001, respectively. For (C) and (H), invasion areas were quantified from three biological replicates. *P*-values were calculated using Student's *t*-test; *, **, and *** denote *P*-value < 0.05, 0.01, and 0.001, respectively.

While some previous studies suggested that PPARG facilitates trophoblast migration and invasion [20, 60], others reported opposite results [16–18]. As PPARG expression showed a modest elevation from TSC to EVT differentiation, with similar *in vivo* expression patterns (Fig. 1A, B; Supplementary Fig. S1A–D), we sought to determine if PPARG is required for EVT differentiation. Upon shRNA-mediated KD in combination with EVT differentiation, we first confirmed that the KD cells displayed impaired EVT differentiation, as observed by their inability to develop the mesenchymal villi morphology (Fig. 3A). Continuous PPARG KD during differentiation was monitored by the reduced protein, and RT-qPCR confirmed the inability to up-regulate EVT markers such as HLA-G, ITGA1, and PLAC8 upon the depletion of PPARG during EVT differentiation (Fig. 3B). Given the crucial role of EVTs in penetrating the maternal uterus, we evaluated how PPARG depletion affects the invasion ability of EVTs using invasion assays like those we have performed previously [27]. The cells with PPARG depletion showed a notably reduced capability to invade the Matrigel barrier (Fig. 3C), confirming that PPARG is necessary for the proper activation of EVT-specific genes and invasive function.

To obtain a transcriptome-level understanding of the defective EVT differentiation by PPARG KD, we conducted RNA-seq and confirmed an impaired activation of EVT markers, HLA-G, ITGA1, and PLAC8, consistent with our RT-qPCR results (Fig. 3D). Moreover, we found that recently identified TFs required for EVT functions, including DLX5, DLX6, and ZNF439 [42], are not properly activated during EVT differentiation upon PPARG KD (Fig. 3D). This trend of impairment was more evident when we performed GSEA with the EVT-specific gene set obtained from human placental scRNA-seq [55] (Fig. 3E). Accordingly, defective down-regulation of TSC markers (TEAD4, MSX2, and TP63) was observed in PPARG KD cells (Fig. 3D). GSEA with the VCT gene set also confirmed that these genes are not properly down-regulated in EVTs with PPARG depletion (Fig. 3E). The genes showing lower expression levels in PPARG KD cells were enriched in GO terms associated with important EVT features, such as extracellular matrix, cell motility, and extracellular matrix proteoglycans (Fig. 3F). On the other hand, the genes linked to the terms associated with TSC self-renewal, such as Hippo signaling pathway and cell population proliferation, were exclusively active in PPARG-depleted EVTs (Fig. 3F). All these findings indicate that PPARG is crucial for EVT differentiation, and its depletion results in impaired EVT differentiation.

Since DLX6, a TF required for late-stage EVT differentiation [42], is significantly down-regulated by PPARG KD (Fig. 3D), we investigated the functional relationship between PPARG and DLX6. Ectopic expression of DLX6 partially rescued the branching morphology, invasive capacity, and expression of EVT markers in PPARG-depleted cells (Supplementary Fig. S4A–D). These results suggest that DLX6 functions downstream of PPARG and mediates its role in EVT differentiation in part.

### PPARG promotes EVT differentiation through ligand-sensitive transcriptional mechanisms

To further investigate the molecular mechanism of PPARG in EVT differentiation, we attempted to disrupt the LBD of PPARG with T007 from day 1 of EVT differentiation until day 8. Upon T007 treatment, cells exhibited an impaired transition to mesenchymal, spindle-like morphology starting on day 3, compared with control EVT differentiation, even at a very low concentration (0.3 μM) (Fig. 3G; Supplementary Fig. S3H). The invasion assay with T007-treated cells resulted in impaired invasion, as evidenced by defects in penetrating the Matrigel (Fig. 3H), similar to those observed in PPARG KD (Fig. 3C). Consistent with these phenotypes, the expression levels of EVT markers were not activated in T007-treated cells (Fig. 3I). Together with the defects observed upon PPARG KD, these findings reinforce that PPARG is essential for EVT differentiation, and functions through LBD-mediated transcriptional activation.

Considering that the phenotypic impairment caused by T007 started to appear on day 3 of EVT differentiation, the time point when mesenchymal morphology typically appears (Fig. 3G), we conducted RNA-seq for the T007-treated samples on EVT day 3 to investigate short-term effects in addition to endpoint samples (EVT day 8) for assessing long-term effects. The results indicated that the T007-treated cells had difficulties in activation of most EVT-specific genes identified from scRNA-seq of early human placenta [55] compared with the control, as observed in the results from both day 3 and day 8 (Supplementary Fig. S4E). GO term analysis of day 8 data revealed that genes up-regulated in T007-treated cells were enriched in processes related to cell growth and the cell cycle, including mitotic cell cycle progression, proliferation, and DNA damage response (Supplementary Fig. S4F). On the other hand, genes that are known to play crucial roles in EVT differentiation, such as those associated with migration and extracellular matrix organization, are less active compared with the control cells (Supplementary Fig. S4F). Furthermore, GSEA revealed that genes down-regulated by T007 treatment are enriched in the EVT-specific gene set, whereas gene sets associated with VCT and ST are more active in T007-treated cells (Supplementary Fig. S4G). Together, these findings demonstrate that inhibition of LBD-mediated transcriptional activity recapitulates key features of PPARG KD, supporting a ligand-sensitive role for PPARG in EVT differentiation.

To additionally define when PPARG activity becomes ligand-sensitive, we treated cells with the selective antagonist T007 at various time points during EVT differentiation (days 0, 1, 2, 3, 4, and 6) and assessed the changes in cell morphology and the transcriptome on day 8. Early T007 treatment (day 0 or 1) resulted in severe morphological defects and substantial transcriptomic changes compared with control EVTs, including significantly reduced EVT gene expression [42]. In contrast, later T007 addition (day 4 or 6) showed significantly milder effects, suggesting that ligand-sensitive transcriptional activity is particularly critical during the early stages of EVT differentiation (Supplementary Fig. S5A–C). Additionally, by monitoring the global target occupancy patterns of PPARG during the time-course of EVT differentiation, we found that PPARG binding in TSCs is clearly distinct from all EVT stages, with a rapid change in chromatin occupancy occurring as early as day 2 (Supplementary Fig. S5D). For example, at key EVT genes such as ITGA1 and DLX6, we observed strong PPARG binding starting from day 2, which persisted throughout differentiation (Supplementary Fig. S5E). Together, these findings indicate a rapid functional shift in PPARG activity during EVT differentiation, with increasing reliance on LBD-mediated transcriptional activation, as evidenced by early T007 sensitivity and dynamic changes in chromatin occupancy.

### Agonist-induced PPARG hyperactivation enhances EVT invasion

**Figure 4.**
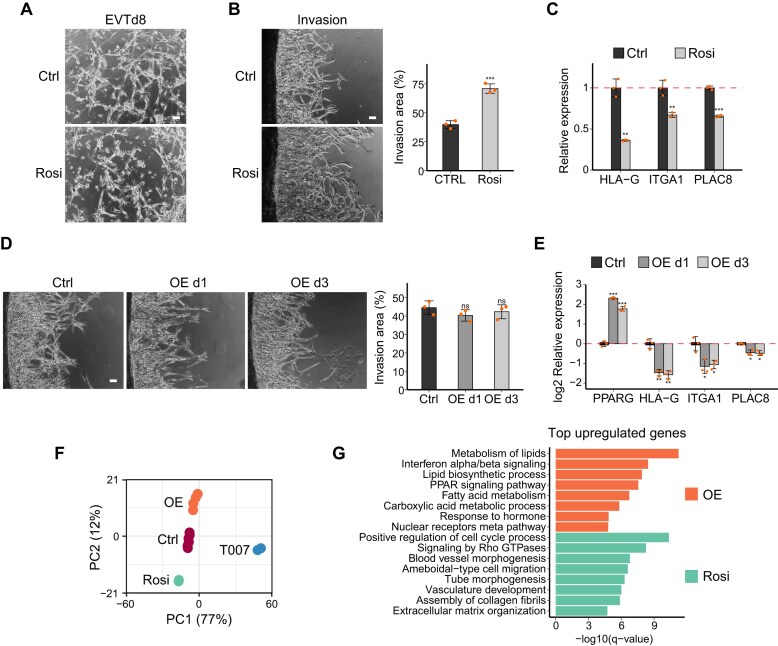
Agonist-induced PPARG activation, rather than elevated protein abundance, enhances EVT invasion. (**A**) Brightfield images of control and Rosi-treated EVTs on day 8 (scale bar = 100 μm). (**B**) Invasion assay of control and Rosi-treated EVTs on day 7 (scale bar = 100 μm). (**C**) Relative expression levels of EVT marker genes in Rosi-treated EVTs compared with control. (**D**) Invasion assay of control and PPARG OE EVTs on day 8 (scale bar = 100 μm). OE d1: overexpression initiated on day 1 of differentiation and sustained until terminal differentiation. OE d3: overexpression initiated on day 3 of differentiation and sustained until terminal differentiation. (**E**) Relative expression levels of EVT marker genes in PPARG OE EVTs compared with control. *P*-values were calculated using Student's *t*-test with three biological replicates; *, **, and *** indicate *P*-value < 0.05, 0.01, and 0.001, respectively. (**F**) Principal component analysis (PCA) showing the overall variation in gene expression profiles among control, T007-treated, Rosi-treated, and PPARG OE EVTs. (**G**) GO enrichment analysis of the top up-regulated genes by PPARG OE or Rosi treatment. For (B) and (D), invasion areas were quantified from three biological replicates. *P*-values were calculated using Student's *t*-test; *, **, and *** denote *P*-value < 0.05, 0.01, and 0.001, respectively; ns, not significant.

PPARG has been implicated in PE, a symptom with shallow invasion due to defective EVT differentiation, which was recapitulated by KD or T007 treatment (Fig. 3; Supplementary Fig. S5). To additionally assess PPARG’s role in EVT differentiation and invasion, we determined the impact of PPARG hyperactivation by treating a synthetic PPARG-specific agonist, Rosi [61], or by PPARG overexpression (OE). While Rosi has been suggested as a drug to treat PE [25], some studies resulted in opposite outcomes [19]. Intriguingly, we observed that Rosi-treated EVTs exhibited control EVT-like morphology in 2D culture (Fig. 4A). However, the cells showed even enhanced invasion capability over the control EVTs in our invasion assays (Fig. 4B), suggesting hyperactive PPARG in regulating genes related to the invasiveness of EVTs. Unexpectedly, we observed an impaired activation of the EVT marker genes HLA-G, ITGA1, and PLAC8 by RT-qPCR upon Rosi treatment (Fig. 4C). To further investigate the role of PPARG in EVT invasion, we additionally performed OE of PPARG isoform 1, the predominant placental isoform [62], starting from day 1 or day 3 throughout the EVT differentiation process by employing an SBFB Dox-inducible system [56]. Surprisingly, unlike Rosi treatment, PPARG OE did not significantly enhance EVT invasion ability (Fig. 4D). Notably, RT-qPCR analysis revealed that while showing normal-like invasiveness, PPARG OE causes an imbalance in gene expression, disrupting the normal EVT expression profile (Fig. 4E). The results reveal that an aberrant overinvasion phenotype of EVTs can be achieved by PPARG hyperactivation via Rosi but not by PPARG OE.

Enhanced invasion reminded us of placental accreta, a condition occurring when the placenta grows too deeply into the uterine wall [63]. To gain deeper insights into the enhanced invasion via Rosi treatment and the associated pathways, we conducted transcriptome analysis using RNA-seq data from T007-treated, Rosi-treated, PPARG OE, and control EVTs. As expected, PCA showed that T007-treated cells are largely different from the control, PPARG OE, or Rosi-treated cells (Fig. 4F). We then explored the distinct biological pathways influenced by PPARG OE or Rosi treatment. Interestingly, our analysis revealed that the top up-regulated genes by PPARG OE are predominantly involved in lipid metabolism, rather than cell migration or invasion, which is consistent with the results from cell invasion assays (Fig. 4D, G). In contrast, Rosi treatment primarily induced genes associated with blood vessel development and extracellular matrix organization—pathways that are more specific to invasiveness (Fig. 4G). Next, we identified the 216 genes (Supplementary Table S4) up-regulated by Rosi treatment and down-regulated by T007 treatment. These genes, which are probably directly affected by PPARG activity, were significantly enriched in various GO terms associated with EVT-specific processes, including extracellular matrix organization and angiogenesis (Supplementary Fig. S6A, B).

Given that Rosi induces a hyperinvasive phenotype, we investigated whether it could mitigate the effects of reduced PPARG expression. We applied Rosi in a PPARG KD context or in combination with T007, and conducted invasion assays. Interestingly, Rosi-induced PPARG hyperactivation led to a modest, partial recovery in EVT invasion capacity under PPARG KD conditions (Supplementary Fig. S6C). Accordingly, the decreased expression of EVT markers upon PPARG KD was partially rescued by Rosi (Supplementary Fig. S6D). However, Rosi did not counteract the inhibition of PPARG activity by T007 (Supplementary Fig. S6E, F), suggesting that the antagonistic effect of T007 dominates, preventing Rosi from effectively activating PPARG. These findings suggest that while Rosi may promote EVT invasion under conditions of reduced PPARG expression, its effect is compromised when PPARG’s LBD-mediated transcriptional activity is inhibited.

To further assess whether the pro-invasive effect of Rosi is mediated specifically through PPARG activation rather than off-target mechanisms, we tested another PPARG-selective agonist, Troglitazone (Trog), which also binds to the LBD [64]. Consistent with the Rosi results, Trog treatment enhanced EVT invasion in both control and PPARG KD conditions (Supplementary Fig. S6G, H), supporting the interpretation that ligand-activated PPARG contributes to the hyperinvasive phenotype. Moreover, the inability of PPARG OE to induce similar invasion suggests that ligand-induced conformational activation, rather than increased PPARG protein levels alone, is critical for this effect. These findings raise the possibility that aberrant PPARG activation may contribute to pathologically enhanced invasion in conditions such as placenta accreta, especially in the context of a scarred maternal endometrium [65–67].

### Agonist-induced activation of RXRA mitigates EVT defects caused by PPARG inactivation

RXRA is a nuclear receptor that dimerizes with PPARG, forming the PPARG–RXRA complex [68]. The PPARG–RXRA heterodimer recruits distinct transcriptional cofactors depending on their LBD states, such as nuclear receptor co-activators (NCOAs) or nuclear receptor co-repressors (NCORs), playing crucial roles in various cellular processes, including metabolic regulation and adipogenesis [69]. Given the suggested importance of the PPARG–RXRA heterodimer in human placenta development [21, 70], we conducted shRNA-mediated KD of RXRA during EVT differentiation. Similar to PPARG, RXRA was found to be essential for proper EVT differentiation (Supplementary Fig. S7A–C). This is evidenced by abnormal cell morphology, impaired invasion ability, and decreased expression of EVT markers.

**Figure 5.**
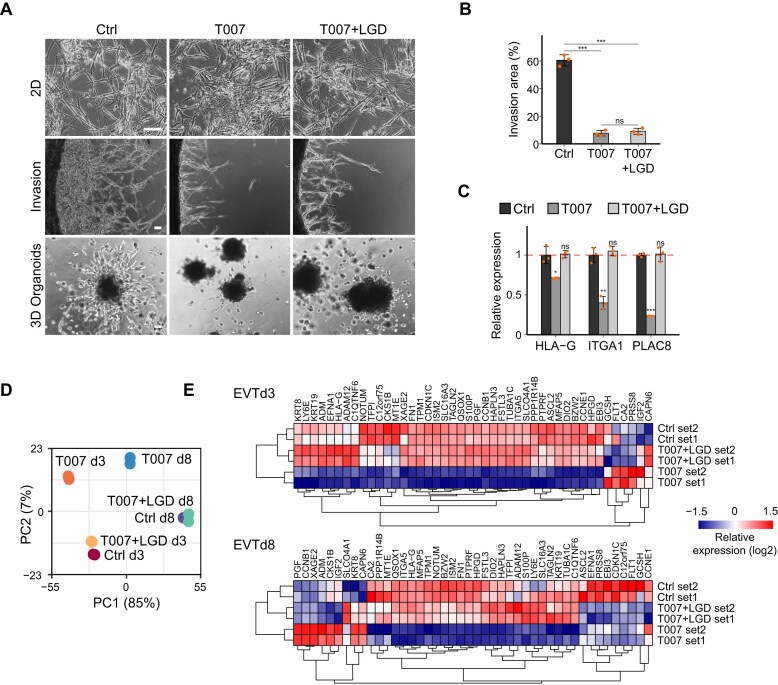
RXRA activation partially rescues the defective EVT differentiation caused by the PPARG antagonist. (**A**) Top: brightfield images showing control, T007-treated, and T007 + LGD-treated EVTs on day 8 (scale bar = 100 μm). Middle: invasion assay of control, T007-treated, and T007 + LGD-treated EVTs on day 8 (scale bar = 100 μm). Bottom: 3D trophoblast organoid showing the EVT invasion capacity under control, T007-treated, and T007 + LGD-treated conditions (scale bar = 100 μm). (**B**) Invasion areas were quantified from three biological replicates. *P*-values were calculated using Student's *t*-test; *, **, and *** denote *P*-value < 0.05, 0.01, and 0.001, respectively; ns, not significant. (**C**) Relative expression levels of EVT markers in cells treated with T007 or T007 combined with LGD compared with control EVTs. *P*-values were calculated using Student's*t*-test with three biological replicates; *, **, and *** indicate *P*-value < 0.05, 0.01, and 0.001, respectively; ns, not significant. (**D**) PCA plot showing the overall variation in gene expression profiles among control, T007-treated, and T007 + LGD-treated cells on EVT day 3 and day 8. (**E**) Heatmap showing the relative expression levels of EVT marker genes identified from scRNA-seq data of human first-trimester placenta in control, T007-treated, and T007 + LGD-treated cells on EVT day 3 (EVTd3) and day 8 (EVTd8).

To gain deeper insights into the functional relationship between PPARG and RXRA during EVT differentiation, we applied the RXR-selective agonist LGD1069 (LGD) [71], together with PPARG antagonist T007, from day 1 of EVT differentiation, and collected samples on day 3 and 8 for expression analysis. As LGD is not RXRA specific, we tested the levels of other RXRs, confirming that RXRA is constantly expressed during EVT differentiation, while RXRB is expressed at approximately a quarter of that level, and RXRG is barely detectable [42] (Supplementary Fig. S7D). While we did not observe a rescue phenotype from combined T007 and Rosi treatment (Supplementary Fig. S6E, F), co-treatment with T007 and LGD partially restored EVT mesenchymal morphology, contrasting with cells treated with T007 only (Fig. 5A, B). Accordingly, LGD reversed the suppression of EVT marker genes (HLA-G, ITGA1, and PLAC8) induced by T007 (Fig. 5C). The impact of the RXRA-selective agonist CD3254 also showed a similar rescue phenotype (Supplementary Fig. S7E, F) [72]. Interestingly, although we observed only a minimally rescued invasion phenotype in 2D culture, a more robust recovery was observed under 3D organoid conditions, showing invasion comparable to that of the control organoids (Fig. 5A). RNA-seq followed by PCA confirmed that the global expression profile of cells co-treated with T007 and LGD more closely resembled that of the control group yet remained distinct from T007-treated cells (Fig. 5D). Importantly, EVT-specific gene expression suppressed by T007 was substantially restored with LGD co-treatment (Fig. 5D, E). These findings suggest that RXRA activation can partially compensate for inhibited PPARG LBD activity, potentially by restoring the transcriptional conformation and function of the PPARG–RXRA complex. This aligns with prior observations in other systems where RXRA ligands were able to rescue compromised heterodimer activity under partial PPARG inhibition [58, 73]. Taken together, these observations identify RXRA agonists as promising candidates for combination therapy in the treatment of PE.

### Integration of hypoxia and ERK pathways in PPARG-mediated EVT differentiation

Given the critical role of PPARG in EVT differentiation and its ligand-sensitive activity, we investigated how extrinsic and intrinsic signals modulate its expression and activity. Recognizing the established importance of oxygen tension in placental development [74, 75], we examined how hypoxic stress affects PPARG-mediated transcriptional regulation during EVT specification. We found that hypoxic stress, modeled by dimethyloxalylglycine (DMOG)-induced stabilization of HIF1A [76], impairs both PPARG expression and its downstream transcriptional activity during EVT differentiation. Notably, DMOG treatment also reduced protein levels of GCM1, a TF critical for ST and EVT differentiation [77] and impaired EVT invasion (Supplementary Fig. S8A, B). This aligns with previous studies in mice, which suggest that Pparg promotes Gcm1 expression to support trophoblast differentiation [78] and that HIF1A represses GCM1 under hypoxic conditions [79]. Transcriptome analysis further revealed a general down-regulation of EVT-specific genes, resembling the defects observed with PPARG depletion (Supplementary Fig. S8C). Additionally, we identified a shared set of DEGs that were significantly regulated in both conditions and performed correlation analysis. The scatter plot revealed a strong positive correlation (Pearson *r* = 0.69), indicating that DMOG and PPARG KD perturb similar sets of genes (Supplementary Fig. S8D). Integrative analysis of DMOG-induced transcriptional changes and PPARG occupancy demonstrated significant overlap between genes down-regulated by hypoxia and PPARG targets (Supplementary Fig. S8E), indicating that the hypoxic microenvironment may hinder EVT differentiation, in part by attenuating the PPARG-driven transcriptional program.

Additionally, we explored intracellular signaling cascades involved in PPARG-dependent EVT differentiation. GO enrichment analysis of genes down-regulated upon PPARG KD revealed several invasion-associated pathways (highlighted in red) [80–84] (Supplementary Fig. S8F). Based on their established roles in trophoblast biology and availability of phospho-protein markers, we focused on PI3K-Akt and MAPK/ERK for experimental validation. We found that PPARG depletion selectively reduces ERK1/2 phosphorylation without significantly affecting AKT phosphorylation (Supplementary Fig. S8G). These results suggest that the MAPK/ERK pathway may serve as a crucial mediator of PPARG-dependent EVT differentiation. Consistently, inhibition of ERK signaling using the selective inhibitor SCH772984 (SCH) [85] resulted in decreased EVT invasion and down-regulation of EVT-specific genes (Supplementary Fig. S8H, I). Together, these findings highlight the diverse roles of PPARG in integrating extrinsic and intrinsic signals to modulate EVT-specific transcriptional programs.

### PPARG-centric transcriptional networks in trophoblast cell types

As PPARG occupies common and unique targets in TSCs and EVTs with enriched motifs of TFs previously implicated in TSC self-renewal and EVT differentiation (Fig. 1C; Supplementary Fig. S2A), we hypothesized that PPARG may form human trophoblast cell-type-specific regulatory circuitries with cell-type-specific TFs, modulating TSC- or EVT-specific cellular functions via controlling numerous downstream target genes. Given the distinct targets of PPARG mapped in TSCs and EVTs (Fig. 1C, D) and a few recent studies revealing key TFs controlling TSC self-renewal and EVT differentiation [42, 53], we sought to test whether PPARG shares its targets with previously defined TFs implicated in TSCs and EVTs. Considering the known interaction between PPARG and RXRA [68, 86], RXRA’s role in EVT differentiation (Supplementary Fig. S7A–C), and the RXR motif enriched within PPARG target loci (Supplementary Fig. S2A), we additionally performed ChIP-seq of RXRA in TSCs and EVTs. As shown, the binding patterns of PPARG and RXRA are highly similar in both TSCs and EVTs (Supplementary Fig. S9A), consistent with their reported dimerization in other cellular contexts [87, 88].

**Figure 6.**
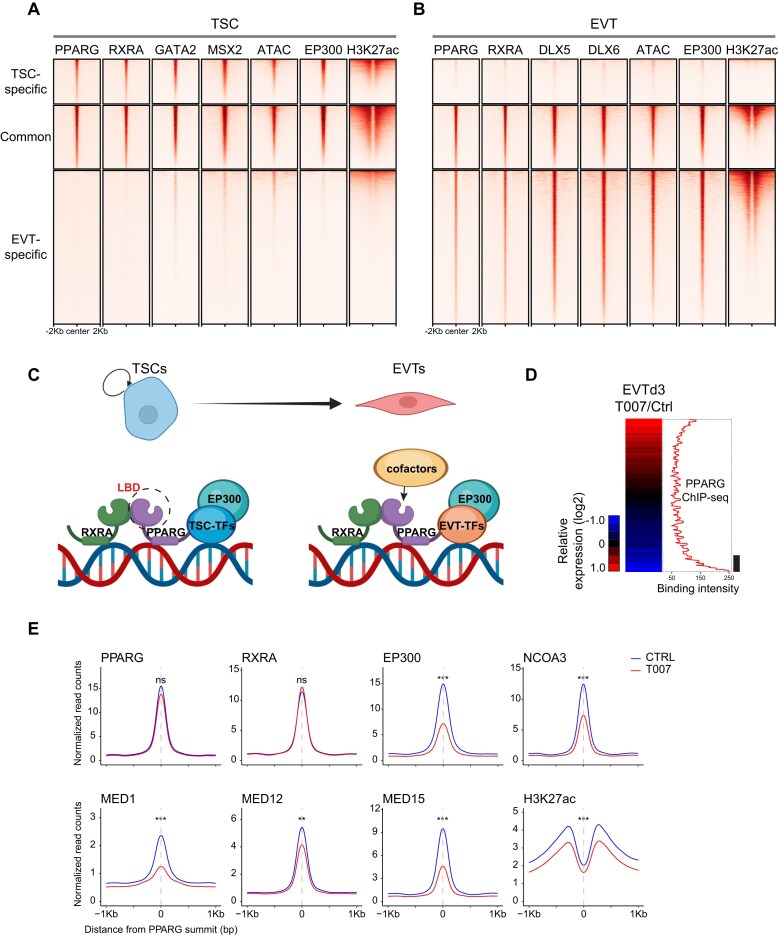
PPARG-centric transcriptional networks with cell-type-specific TFs and cofactors. (**A**) Heatmap showing the ChIP-seq signals of PPARG, RXRA, TSC TFs (MSX2 and GATA2), EP300, and H3K27ac, along with ATAC-seq signals at PPARG-bound TSC-specific, common, and EVT-specific loci in TSCs. (**B**) Heatmap showing the ChIP-seq signals of PPARG, RXRA, EVT TFs (DLX5 and DLX6), EP300, and H3K27ac, along with ATAC-seq signals at PPARG-bound TSC-specific, common, and EVT-specific loci in EVTs. (**C**) Model: in TSCs, PPARG associates with RXRA and TSC-specific TFs to maintain self-renewal independently of LBD-mediated transcriptional activity. In contrast, during EVT differentiation, PPARG cooperates with RXRA, LBD-associated cofactors, and EVT-specific TFs to activate gene expression via LBD-mediated transcriptional regulation. (**D**) Heatmap showing the log2-normalized gene expression upon T007 treatment on EVT day 3 (left). Genes were ordered according to their relative expression levels in T007-treated cells versus control. The PPARG occupancy signals on EVT day 3 for these ordered genes were plotted as the moving window average (window size, 100; bin size, 1) (right). (**E**) Histograms showing ChIP-seq read enrichment for PPARG, RXRA, EP300, NCOA3, MED1, MED12, MED15, and H3K27ac in control cells (blue) and T007-treated cells (red) on EVT day 3, around selected PPARG summits associated with genes significantly down-regulated by T007. Data are shown as mean normalized read counts from two biological replicates. Statistical comparisons were performed using two-tailed Student's *t*-tests; *, **, and *** indicate *P*-value < 0.05, 0.01, and 0.001, respectively; ns, not significant.

To explore the PPARG-centered transcriptional networks during trophoblast development, we analyzed our newly generated and published ChIP-seq data in TSCs and EVTs [42, 52, 53]. As shown in Fig. 6A and B, integrated heatmaps revealed PPARG occupancy patterns in TSCs and EVTs, along with occupancy signals from RXRA, TSC-specific (GATA2 and MSX2) and EVT-specific (DLX5 and DLX6) TFs, as well as the H3K27ac enhancer histone mark, EP300 binding, and chromatin accessibility. The results suggest that in TSCs, PPARG collaborates with RXRA, GATA2, and MSX2, co-occupying many common targets. In EVTs, PPARG seems to co-regulate its targets with RXRA, DLX5, and DLX6 by forming distinct transcriptional regulatory circuits. The cell-type-specific occupancy patterns of PPARG and other TSC- or EVT-specific TFs are well aligned with the active enhancer marker H3K27ac. Additionally, the chromatin accessibility patterns are also in line with the target occupancy patterns of PPARG in TSCs and EVTs (Fig. 6A, B). These analytical results suggest that during EVT differentiation, transcriptional re-wiring of multiple TFs happens. As exemplified, the EVT marker ITGA1 was co-occupied by PPARG, RXRA, DLX5, and DLX6 in EVTs, but not in TSCs (Supplementary Fig. S9B), supporting that PPARG engages in a dynamic regulatory network with EVT-specific TFs in EVTs (Fig. 6C).

To evaluate how T007 treatment, which impairs EVT differentiation (Fig. 3G–I; Supplementary Fig. S5A–C), affects the PPARG-centric regulatory network, we monitored changes in the binding patterns of PPARG and PPARG-associated factors, including RXRA, EP300 and NCOA3, as well as H3K27ac on EVT differentiation day 3 upon T007 treatment. Given the role of nuclear receptors in mediator recruitment for enhancer activation [89, 90], we also explored the binding of mediator components MED1, MED12, and MED15. We observed that PPARG, RXRA, and these cofactors exhibited extensive co-occupancy at shared genomic regions (Supplementary Fig. S9C). In addition, our combined analysis of transcriptome changes upon T007 treatment and target occupancy of PPARG on day 3 of EVT differentiation revealed that the genes not properly up-regulated upon T007 treatment are directly controlled by PPARG (Fig. 6D). Focusing on the PPARG loci near these genes (Supplementary Table S5), we monitored the changes in the ChIP-seq signals and found that the occupancy signals of EP300, NCOA3, MED1, MED12, MED15, and H3K27ac are reduced, whereas PPARG and RXRA binding remain relatively stable (Fig. 6E). For instance, although the binding signals of PPARG and RXRA remained comparable, the binding of all cofactors diminished upon T007 treatment near the EVT-associated genes, such as DLX5 and WWTR1 (Supplementary Fig. S9D). These results suggest that the EVT-specific regulatory network can be disturbed by T007 via interfering with cofactor recruitment to the target loci, without significantly affecting the DNA binding affinity of PPARG and RXRA.

To further assess the physical associations between PPARG and TFs or cofactors at the protein level, we performed co-IP followed by western blot. On EVT day 3, PPARG is associated with EP300, the mediator subunits MED1/MED15, and its known dimerization partner RXRA (Supplementary Fig. S9E). Notably, we also observed cell-type-specific association patterns: in TSCs, PPARG is co-precipitated with MSX2, whereas in EVTs, it is associated with DLX5 (Supplementary Fig. S9F, G), consistent with PPARG’s cell-type-specific co-occupancy patterns identified by ChIP-seq (Fig. 6A, B). Overall, our findings illustrate that PPARG contributes to distinct transcriptional networks in TSCs and during EVT differentiation by establishing distinct transcriptional networks with cell-type-specific TFs. The involvement of additional cofactors further underscores the complexity of PPARG-mediated regulatory networks, highlighting its important role in modulating gene expression and functions specific to trophoblast cell types.

## Discussion

In the current study, we employed the human TSC model to investigate the roles of PPARG in TSC self-renewal and EVT differentiation, and thereby in human trophoblast lineage development. Our results suggest that PPARG is required for proper maintenance of TSC identity and promotion of EVT differentiation. Specifically, we found that EVT differentiation depends on the LBD-mediated transcriptional activity of PPARG and involves PPARG-centric transcriptional re-wiring. Notably, PPARG functions through distinct mechanisms in TSCs and EVTs, with differential reliance on LBD-mediated activity. These findings reveal a dual mode of PPARG action: it supports TSC self-renewal through a ligand-insensitive mechanism, whereas EVT differentiation relies on ligand-sensitive, LBD-mediated transcriptional activation, highlighting the context-specific importance of LBD function in trophoblast cell fate transitions.

Notably, while our data show that PPARG is not highly expressed in STs, some prior studies indicated that PPARG plays a critical role in ST differentiation and enhances the expression level of the ST marker CGB [22]. While we do not directly address PPARG’s role in ST lineage specification in the current study, considering its relatively lower expression in STs both *in vivo* and *in vitro*, proper down-regulation of PPARG may be critical for normal ST differentiation.

Despite its crucial roles suggested in the trophoblast lineage, the chromosomal targets of PPARG in human trophoblasts are not known. We identified previously unreported chromosomal targets of PPARG and RXRA in TSCs and EVTs. In TSCs, PPARG and RXRA co-bind TSC-specific loci with other known TSC TFs, whereas in EVTs, PPARG and RXRA co-occupy EVT-specific loci with EVT TFs, collectively modulating cell-type-specific gene expression patterns among human trophoblast subtypes. Additionally, we performed co-IP to identify PPARG-associated proteins in TSCs and EVTs, confirming the existence of distinct, cell-type-specific regulatory complexes. By integrating ChIP-seq data for TFs, cofactors, mediator subunits, and the active enhancer mark H3K27ac, we demonstrate that PPARG functions within dynamic regulatory networks to modulate enhancer activity in a context-dependent manner.

Our results also have implications for understanding pregnancy complications such as PE and placenta accreta. Previous research has suggested a potential link between abnormal PPARG expression and PE, a condition characterized by insufficient EVT invasion, potentially affecting placental implantation and maternal spiral artery remodeling [16–18, 91]. Genetic studies have also suggested that variations in the PPARG gene may increase the risk of developing PE [92]. PPARG’s anti-inflammatory properties and role in regulating oxidative stress response suggest that its reduction leads to heightened inflammation and oxidative stress [93, 94]. We confirmed PPARG’s pivotal role in governing EVT invasion and additionally showed that T007-induced perturbation of PPARG may modulate EVT invasiveness in a manner potentially relevant to PE [60, 95]. Moreover, our results further suggest that RXRA agonists could partially reverse defective invasion phenotypes caused by PPARG dysfunction, raising the possibility of combined modulation of PPARG and RXRA activity in future therapeutic strategies. Our results also suggested that placenta accreta, another severe pregnancy complication characterized by excessive EVT invasion into the uterine wall [63, 65], may also be associated with PPARG dysfunction. Obesity has been reported as a potential risk factor for placenta accreta [96], and we hypothesize that this could be attributed to the close relationship between lipid metabolism and PPARG expression and function. Notably, we found that the PPARG agonist Rosi enhances the invasiveness during normal EVT differentiation, while PPARG OE did not. The distinct effects of PPARG OE and Rosi treatment indicate that EVT invasion is not solely determined by PPARG abundance but rather depends on LBD-mediated transcriptional activity. This underscores the importance of LBD integrity, ligand availability, or LBD-associated cofactor interactions in modulating PPARG function under pathological conditions such as PE or placenta accreta. Further research focused on identifying endogenous factors that affect PPARG activity will provide valuable insights into the mechanisms underlying pregnancy-related complications and potential therapeutic strategies.

In summary, our findings highlight PPARG as a crucial factor for both TSC self-renewal and EVT differentiation. We have identified PPARG-targeted loci and uncovered previously unknown PPARG-involved transcriptional regulatory networks that play critical roles in trophoblast progenitor maintenance and EVT differentiation. Importantly, the dynamic activation state of the PPARG LBD significantly influences gene regulation essential for trophoblast development and invasion. Our research provides valuable insights into the future investigation of PPARG dysregulation in placenta-related diseases, including the underlying mechanisms and potential therapeutic approaches.

## Supplementary Material

gkaf669_Supplemental_Files

## Data Availability

Raw and processed ChIP-seq and RNA-seq data generated in this study have been deposited in the GEO database under accession numbers GSE278374, GSE278375, GSE295568, and GSE295569. The previously published PPARG ChIP-seq data in adipocytes were obtained from GSE64458. The MSX2 ChIP-seq data in TSCs were obtained from GSE165970. The GATA2 ChIP-seq data in TSCs were obtained from GSE208539. The DLX5 and DLX6 ChIP-seq data in EVTs, ATAC-seq data from TSCs and EVTs, and time-course RNA-seq data in TSCs and during differentiation were obtained from GSE212267.
