# Supplementary material for: PPARG-centric transcriptional re-wiring during differentiation of human trophoblast stem cells into extravillous trophoblasts
Source: Nucleic Acids Res. 2025 Jul 24;53(14):gkaf669. doi: 10.1093/nar/gkaf669 (PMC12288872; doi:10.1093/nar/gkaf669)
Supplement: gkaf669_Supplemental_Files [file gkaf669_supplemental_files.zip › Supplementary Data_20250703_v3.pdf]

Supplementary Data

Supplementary Table Legends

Supplementary Table S1. Oligo sequences of RT-qPCR primers and shRNAs
Supplementary Table S2. Grouped PPARG binding loci in TSCs and EVTs
Supplementary Table S3. The differentially expressed genes (DEGs) upon PPARG KD in TSCs (cutoff: |log2FC| > 1, adjusted P-value < 0.05)
Supplementary Table S4. The DEGs upregulated by Rosi treatment overlapped with those downregulated by T007 treatment on EVT day 8 (cutoff: |log2FC| > 1, adjusted P-value < 0.05)
Supplementary Table S5. PPARG binding loci associated with the genes downregulated by T007 on EVT day 3

Supplementary Figure Legends

Supplementary Figure S1. Characterization of PPARG expression patterns in trophoblast cell types.
(A) Violin plot showing the expression levels of CT marker TP63, EVT marker HLA-G, ST marker CGB3, and PPARG across all trophoblast cell subtypes.
(B) UMAP visualization of trophoblast cell clusters from snRNA-seq analysis.
(C) Western blot analysis showing PPARG expression levels in TSCs, EVTs, and STs.
(D) Time-course RNA-seq data illustrating the dynamic changes in PPARG expression during EVT and ST differentiation.
(E) Immunofluorescence staining of PPARG (magenta) in self-renewing TSCs and terminally differentiated EVTs and STs, with DAPI staining (blue) to mark cell nuclei.
(F) Proportion of annotated genomic features of PPARG binding regions in either TSCs or EVTs.

Supplementary Figure S2. Analysis of distinct PPARG binding patterns in trophoblast cell types and adipocytes.
(A) Top de novo motifs enriched at PPARG binding loci in either TSCs or EVTs. Motif analysis was performed using the findMotifsGenome.pl program of HOMER.
(B) Venn diagram of the genes targeted by PPARG in TSCs and adipocytes.
(C) Correlation heatmap displaying the similarities of PPARG binding loci among TSCs, EVTs, and adipocytes.
(D) GO enrichment analysis of PPARG-binding sites specific to TSCs or adipocytes.
(E) Crystal violet staining was used to assess colony formation in TSCs under control and PPARG KD conditions. Colony area was quantified using ImageJ. Statistical significance was determined by Student's t-test (n = 3); *, **, and *** indicate P-value < 0.05, 0.01, and 0.001, respectively.
(F) CCK-8 proliferation assay comparing TSCs under control and PPARG KD conditions. OD450 was measured daily over four days. P-values were calculated using Student's t-test with three biological replicates; *, **, and *** indicate P-value < 0.05, 0.01, and 0.001, respectively.
(G) Heatmap showing the relative expression levels of super-enhancer (SE)-associated TFs in PPARG KD TSCs compared to control TSCs.

Supplementary Figure S3. Validation of PPARG KD specificity and limited sensitivity to T007 in TSCs.

(A) Western blot showing dose-dependent induction of exogenous, shRNA-resistant PPARG upon Dox treatment (12.5 or 15 ng/mL) in TSCs. Left: non-targeting control; right: PPARG KD.
(B) Phase-contrast images showing colony morphology of TSCs under control, PPARG KD, and rescue conditions (scale bar = 100 µm).
(C) CCK-8 proliferation assay comparing TSCs under control, PPARG KD, and rescue conditions (Dox 12.5 or 15 ng/mL). OD450 was measured daily over four days. P-values were calculated using Student's t-test comparing the PPARG KD and rescue groups, with three biological replicates; *, **, and *** indicate P-value < 0.05, 0.01, and 0.001, respectively.
(D) Heatmap of log2-normalized expression of TSC markers and cell cycle-related genes across the indicated conditions.
(E) GSEA comparing PPARG KD versus control and Rescue (Dox 12.5 ng/mL) versus KD groups, using VCT marker genes (left) and TSC SE-associated TFs (SE-TFs) (right). Normalized enrichment scores (NES) and FDR values are indicated.
(F) Phase-contrast images of TSCs treated with increasing concentrations of the PPARG antagonist T007 (0, 0.3, 1, 3, 10, and 30 µM) for 72 hours (scale bar = 100 µm).
(G) Heatmap of log2-normalized expression of all DEGs (n = 1884) identified in PPARG KD and T007-treated cells with indicated concentrations.
(H) Phase-contrast images of EVTs treated with increasing concentrations (0, 0.3, and 1 µM) of the PPARG antagonist T007 during differentiation (scale bar = 100 µm).

Supplementary Figure S4. Rescue of the PPARG KD phenotype by DLX6 expression and disruption of EVT differentiation by T007.
(A) Phase-contrast images (left) and invasion assay (right) of EVTs under control, PPARG KD, or KD+DLX6 OE (rescue) conditions (scale bar = 100 µm). Invasion areas were quantified from three biological replicates. P-values were calculated using Student's t-test; *, **, and *** denote P-value < 0.05, 0.01, and 0.001, respectively.
(B) GSEA comparing DLX6 OE (rescue) versus PPARG KD, using an EVT marker gene set identified from first-trimester human placental scRNA-seq.
(C) Sample-wise Pearson correlation heatmap of RNA-seq data across control, PPARG KD, and KD+DLX6 OE groups.
(D) Bar plots showing transcripts per million (TPM) expression of selected EVT marker genes across the three conditions. Bars represent mean ± standard deviation from two biological replicates; t-tests were used for statistical comparisons; *, **, and *** indicate P-value < 0.05, 0.01, and 0.001, respectively; ns, not significant.
(E) Heatmap showing the relative expression levels of EVT marker genes identified from scRNA-seq data of human first-trimester placenta in control and T007-treated cells on EVT day 3 (EVTd3) and day 8 (EVTd8).
(F) GO enrichment analysis of the top downregulated and upregulated genes upon T007 treatment.
(G) GSEA of T007-treated cells compared to control EVTs, utilizing the CT, EVT, and ST marker gene sets identified from scRNA-seq data of human first-trimester placenta.

Supplementary Figure S5. Dynamic regulation of PPARG occupancy and emergence of ligand sensitivity during EVT differentiation.
(A) Phase-contrast images of EVTs differentiated in the presence of 1 µM T007 added at various time points (days 0, 1, 2, 3, 4, or 6), imaged on day 8 (scale bar = 100 µm).
(B) PCA of RNA-seq profiles from T007-treated and control EVTs, with samples colored according to the timing of T007 addition.

(C) Heatmap of log2-normalized TPM expression for a published EVT-specific gene set across T007-treated samples. Values are normalized to the mean of control EVTs.
(D) Pearson correlation heatmap of PPARG ChIP-seq profiles across EVT differentiation time points (TSC and EVT days 2, 4, 6, and 8).
(E) Genome browser tracks showing PPARG binding dynamics at the ITGA1 and DLX6 loci during EVT differentiation.

Supplementary Figure S6. EVT-associated functions are promoted by the activation of the PPARG LBD.
(A) GO enrichment analysis of the genes upregulated by Rosi that overlap with those downregulated by T007.
(B) Heatmap showing the relative expression levels of collagen or angiogenesis-related genes upregulated by Rosi and downregulated by T007, in T007- or Rosi-treated EVTs compared to control.
(C) Invasion assay of EVTs under control, PPARG KD, or PPARG KD with Rosi conditions on day 8 (scale bar = 100 μm).
(D) Relative expression levels of EVT marker genes in cells under PPARG KD or PPARG KD with Rosi conditions compared to control EVTs.
(E) Invasion assay of EVTs under control, T007, and T007 with Rosi treatment conditions on day 8 (scale bar = 100 μm).
(F) Relative expression levels of EVT markers in cells treated with T007 alone or T007 combined with Rosi compared to control EVTs. P-values were calculated using Student's t-test with three biological replicates; *, **, and *** indicate P-value < 0.05, 0.01, and 0.001, respectively; ns, not significant.
(G) Invasion assay of EVTs under control or Trog treatment conditions on day 7 (scale bar = 100 μm).
(H) Invasion assay of EVTs under control, PPARG KD, or PPARG KD with Trog conditions on day 8 (scale bar = 100 μm).
For panels (C), (E), (G), and (H), invasion areas were quantified from three biological replicates. P-values were calculated using Student's t-test; *, **, and *** denote P-value < 0.05, 0.01, and 0.001, respectively; ns, not significant.

Supplementary Figure S7. RXRA is expressed throughout the differentiation of EVTs and is required for their proper development.
(A) Brightfield images showing EVT morphology under RXRA KD and control conditions on EVT day 8 (scale bar = 100 μm).
(B) Western blot analysis showing the reduced RXRA expression by shRNA-mediated KD on EVT day 8. RT-qPCR showing the relative expression levels of RXRA and EVT marker genes in RXRA KD cells compared to control EVTs.
(C) Invasion assay of EVTs under control or RXRA KD conditions on day 8 (scale bar = 100 μm). Invasion areas were quantified from three biological replicates. P-values were calculated using Student's t-test; *, **, and *** denote P-value < 0.05, 0.01, and 0.001, respectively.
(D) TPM-normalized expression levels of RXRA, RXRB, and RXRG during EVT differentiation.
(E) Brightfield images showing control, T007-treated, and T007+CD3254-treated EVTs on day 8 (scale bar = 100 μm).
(F) Relative expression levels of EVT markers in cells treated with T007 or T007 combined with CD3254 compared to control EVTs. P-values were calculated using Student's t-test with three biological replicates; *, **, and *** indicate P-value < 0.05, 0.01, and 0.001, respectively; ns, not significant.

Supplementary Figure S8. EVT differentiation is modulated by hypoxia and ERK signaling via PPARG-associated mechanisms.
(A) Western blot showing protein levels of HIF1A, PPARG, GCM1, and ACTB in EVTs treated with 500 μM DMOG compared to untreated controls.
(B) Phase-contrast image (left) and invasion assay (right) of EVTs on day 8 under control or DMOG treatment conditions (scale bar = 100 μm).
(C) Heatmap showing the relative expression of EVT marker genes identified from first-trimester human placental scRNA-seq in control and DMOG-treated EVTs.
(D) Correlation of DEGs between PPARG KD and DMOG treatment in EVTs. Each point represents a gene significantly altered in both conditions ($|\log2FC| > 1$, adjusted P-value < 0.05). The red line indicates the linear regression fit, with 2D density contours showing the distribution of gene points.
(E) Left: Heatmap of log2-normalized gene expression in DMOG-treated versus control EVTs, with genes ranked by relative expression in the DMOG condition. Right: Corresponding PPARG ChIP-seq signal (EVT day 8) shown as a moving average (window = 100, bin = 1).
(F) Top signaling pathways enriched among genes downregulated upon PPARG KD. Red text highlights pathways known to promote cell migration and invasion.
(G) Phase-contrast image (left) and invasion assay (right) of EVTs on day 8 under control or SCH treatment conditions (scale bar = 100 μm).
(H) Western blot of ERK1/2 and AKT signaling in EVTs under control and PPARG KD conditions. Both phosphorylated (p-) and total protein levels are shown.
(I) Heatmap showing relative expression of EVT marker genes identified from first-trimester human placental scRNA-seq in SCH-treated and control EVTs.
For panels (B) and (H), invasion areas were quantified from three biological replicates. P-values were calculated using Student's t-test; *, **, and *** denote P-value < 0.05, 0.01, and 0.001, respectively.

Supplementary Figure S9. T007 disrupts the role of PPARG–RXRA in EVT differentiation by hindering the recruitment of cofactors.
(A) Correlation heatmap displaying the similarities in binding loci between PPARG and RXRA in both TSCs and EVTs.
(B) Gene track view showing ChIP-seq signals of PPARG and RXRA in TSCs with GATA2 and MSX2, and in EVTs with DLX5 and DLX6, near the EVT marker ITGA1.
(C) Venn diagram of the loci targeted commonly and uniquely by PPARG, EP300, NCOA3, MED12, and MED15 on EVT day 3.
(D) Gene track view displaying ChIP-seq signals of PPARG, RXRA, EP300, NCOA3, MED1, MED12, MED15, and H3K27ac in control cells (blue) and T007-treated cells (red) on EVT day 3, near DLX5 and WWTR1.
(E) Co-IP followed by Western blot on EVT day 3 shows that PPARG interacts with RXRA, EP300, MED1, and MED15.
(F) Co-IP followed by Western blot in TSCs confirms PPARG's interaction with RXRA and MSX2.
(G) Co-IP followed by Western blot in EVTs on day 8 confirms the interaction between PPARG and RXRA, as well as DLX5.

Guo 2024 Figure S1

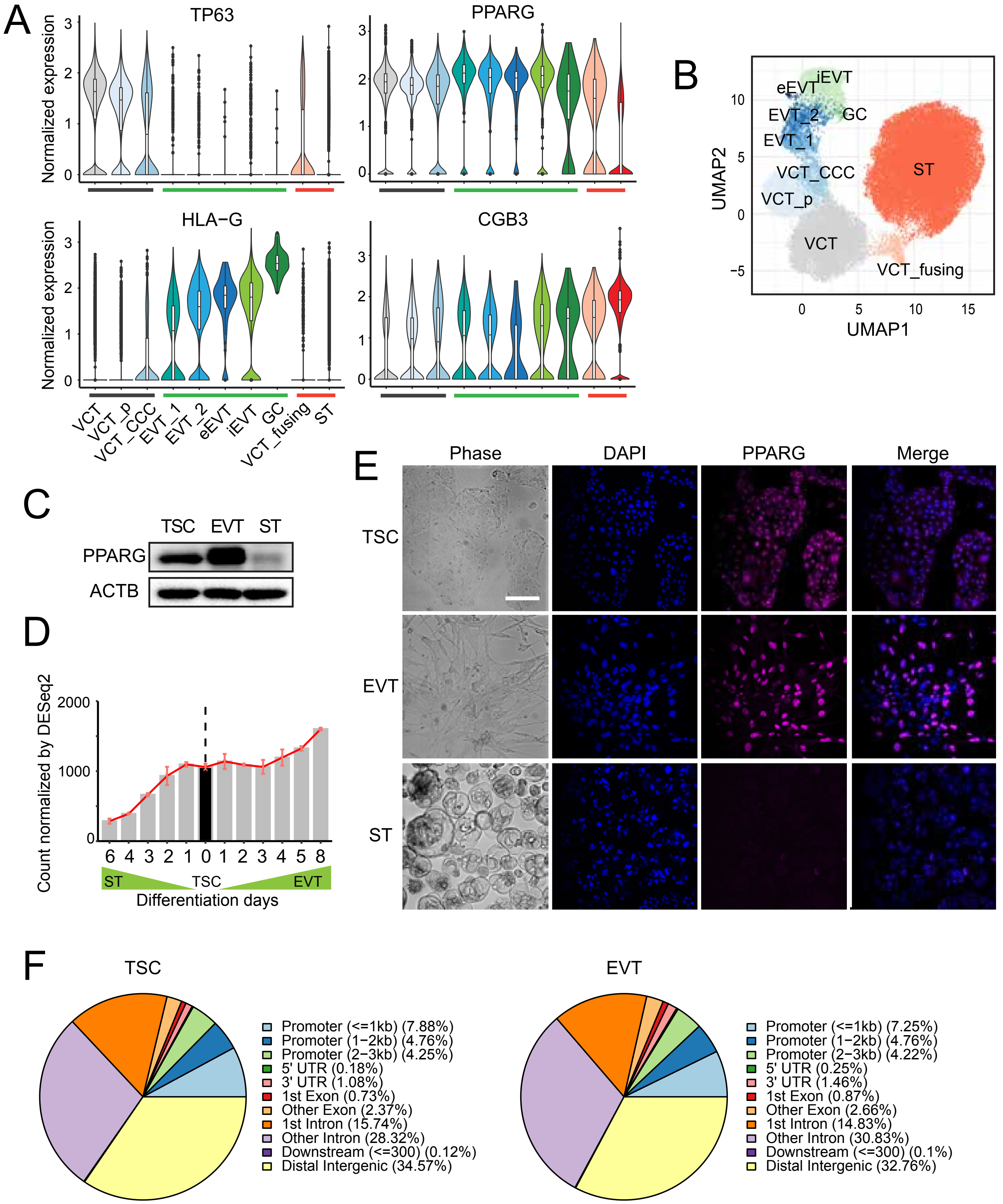

Guo 2024 Figure S2

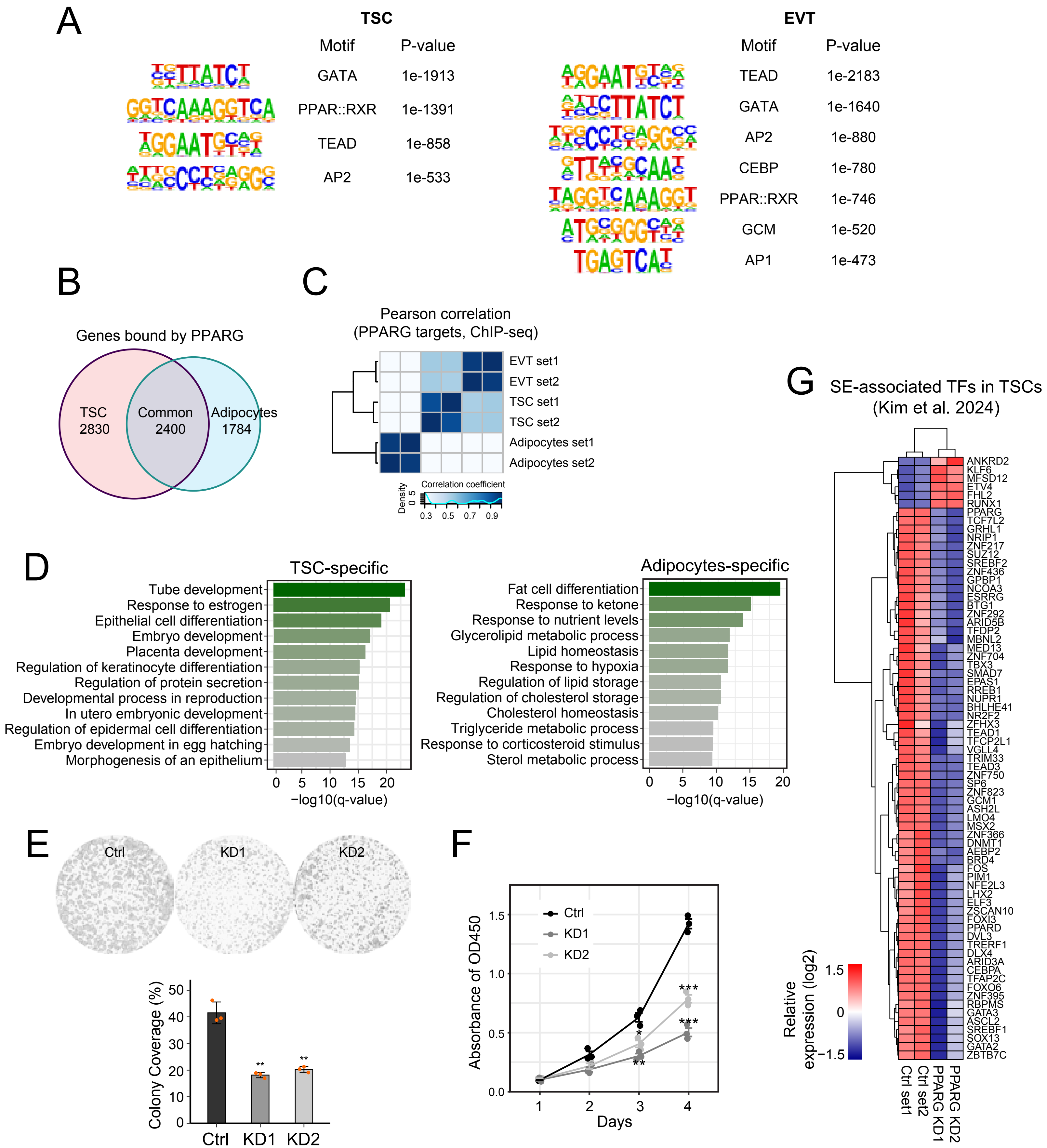

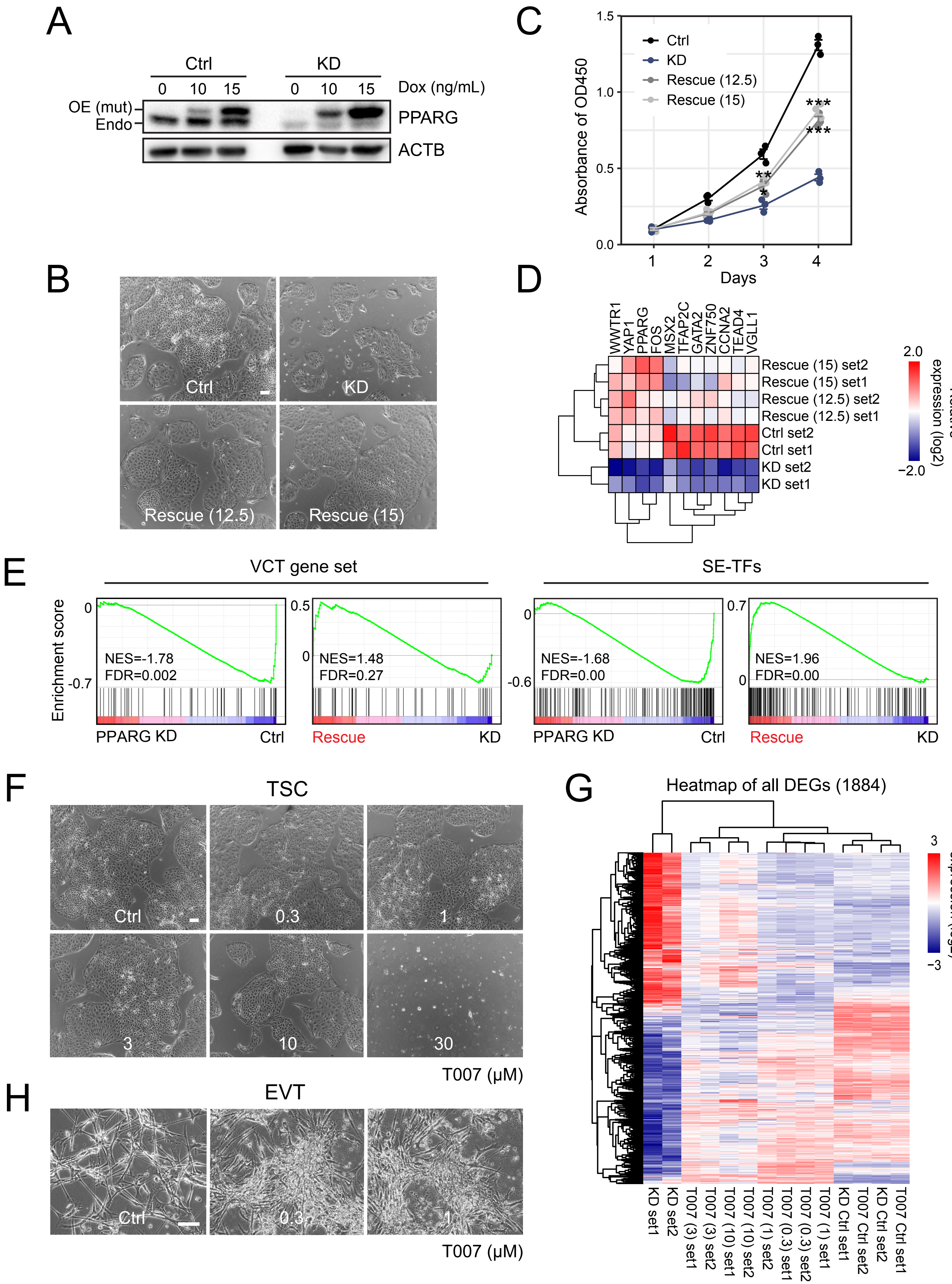
A
Ctrl
KD
0
10
15
Dox (ng/mL)
OE (mut)
Endo
PPARG
ACTB
B
Ctrl
KD
Rescue (12.5)
Rescue (15)
C
Ctrl
KD
Rescue (12.5)
Rescue (15)
Absorbance of OD450
Days
D
WWTR1
YAP1
PPARG
FOS
MSX2
TFAP2C
GATA2
ZNF750
CCNA2
TEAD4
VGLL1
Rescue (15) set2
Rescue (15) set1
Rescue (12.5) set2
Rescue (12.5) set1
Ctrl set2
Ctrl set1
KD set2
KD set1
Relative expression (log2)
E
VCT gene set
SE-TFs
Enrichment score
NES=-1.78
FDR=0.002
NES=1.48
FDR=0.27
NES=-1.68
FDR=0.00
NES=1.96
FDR=0.00
PPARG KD
Ctrl
Rescue
KD
F
TSC
Ctrl
T007 (μM)
G
Heatmap of all DEGs (1884)
Relative expression (log2)
KD set1
KD set2
T007 (3) set1
T007 (3) set2
T007 (10) set1
T007 (10) set2
T007 (1) set2
T007 (0.3) set1
T007 (0.3) set2
T007 (1) set1
KD Ctrl set1
T007 Ctrl set2
KD Ctrl set2
T007 Ctrl set1
H
EVT
Ctrl
T007 (μM)

Guo 2024 Figure S4

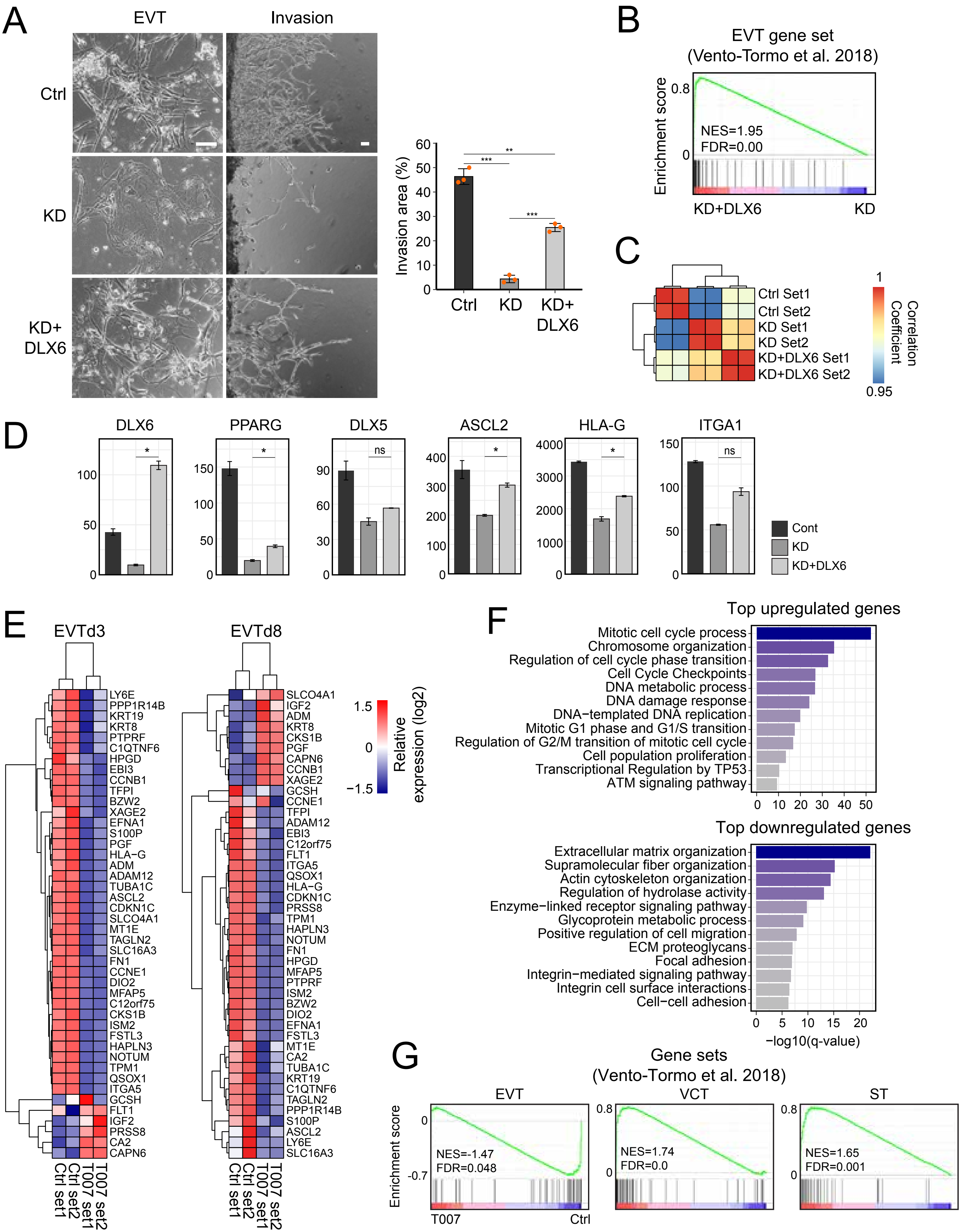

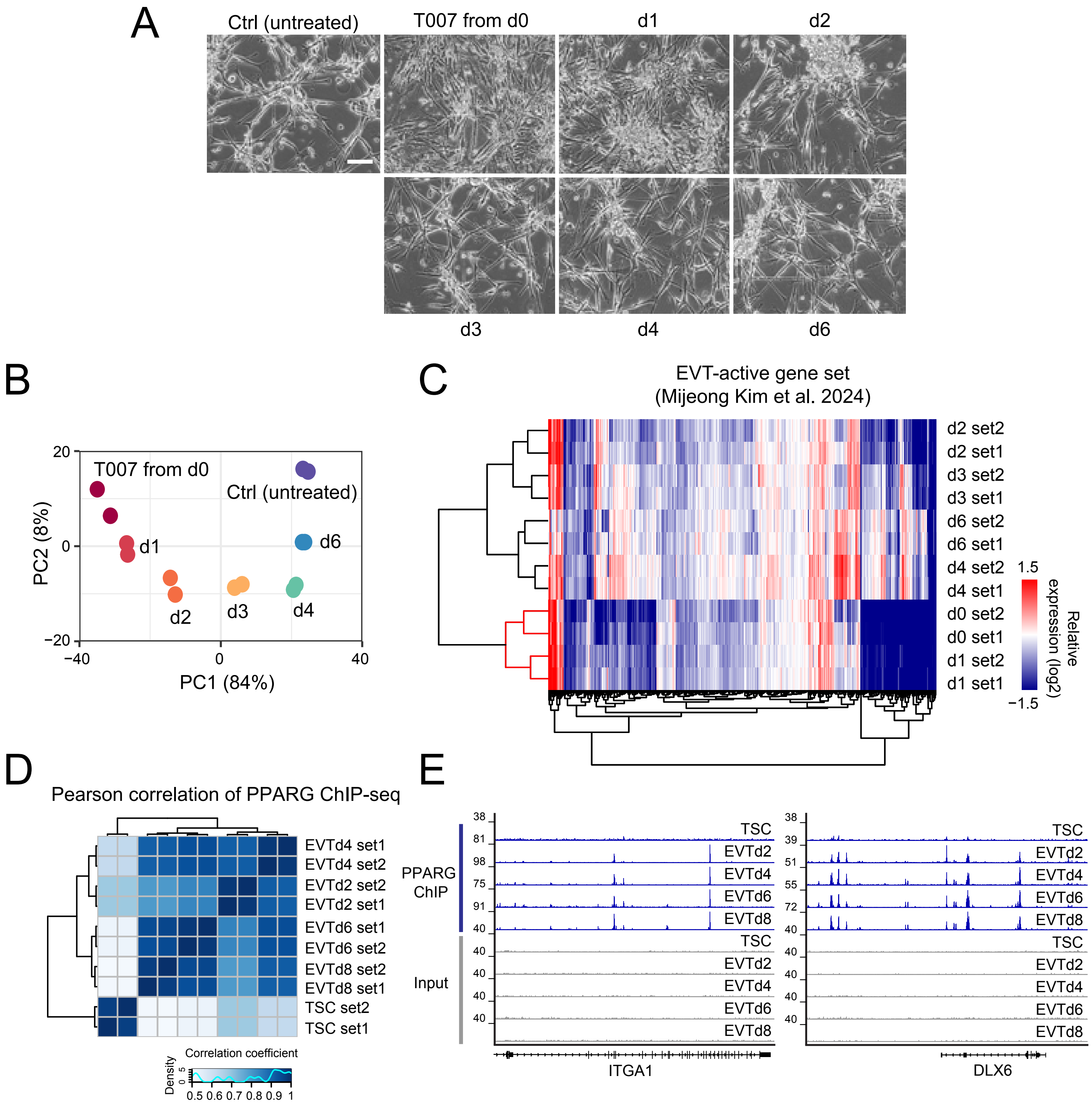

A
Ctrl (untreated)
T007 from d0
d1
d2
d3
d4
d6
B
T007 from d0
Ctrl (untreated)
d1
d6
d2
d3
d4
PC2 (8%)
PC1 (84%)
20
0
−20
−40
0
40
C
EVT-active gene set
(Mijeong Kim et al. 2024)
d2 set2
d2 set1
d3 set2
d3 set1
d6 set2
d6 set1
d4 set2
d4 set1
d0 set2
d0 set1
d1 set2
d1 set1
1.5
−1.5
Relative expression (log2)
D
Pearson correlation of PPARG ChIP-seq
EVTd4 set1
EVTd4 set2
EVTd2 set2
EVTd2 set1
EVTd6 set1
EVTd6 set2
EVTd8 set2
EVTd8 set1
TSC set2
TSC set1
Correlation coefficient
Density
0.5 0.6 0.7 0.8 0.9 1
E
PPARG ChIP
Input
TSC
EVTd2
EVTd4
EVTd6
EVTd8
ITGA1
DLX6

Guo 2024 Figure S6

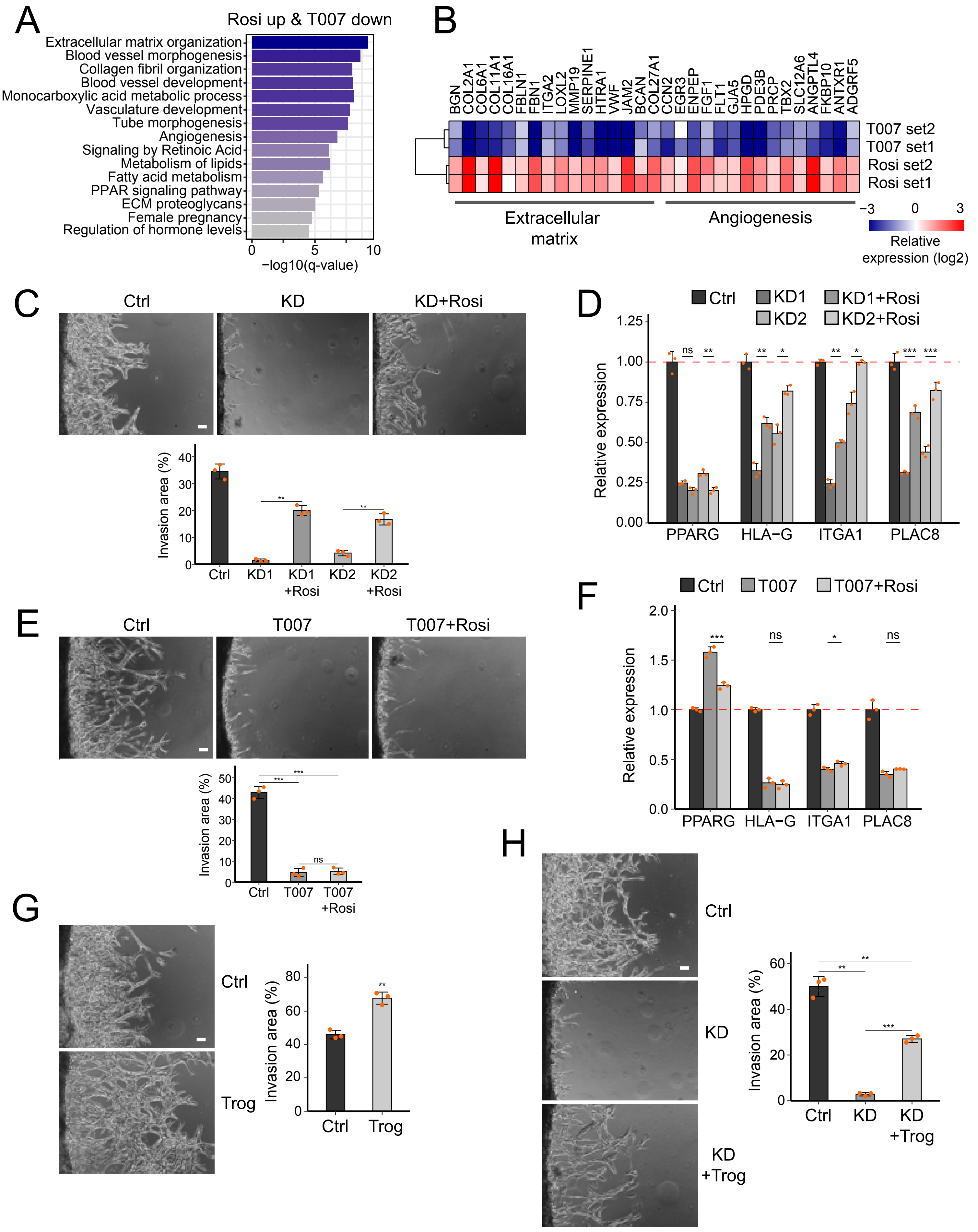

Guo 2024 Figure S7

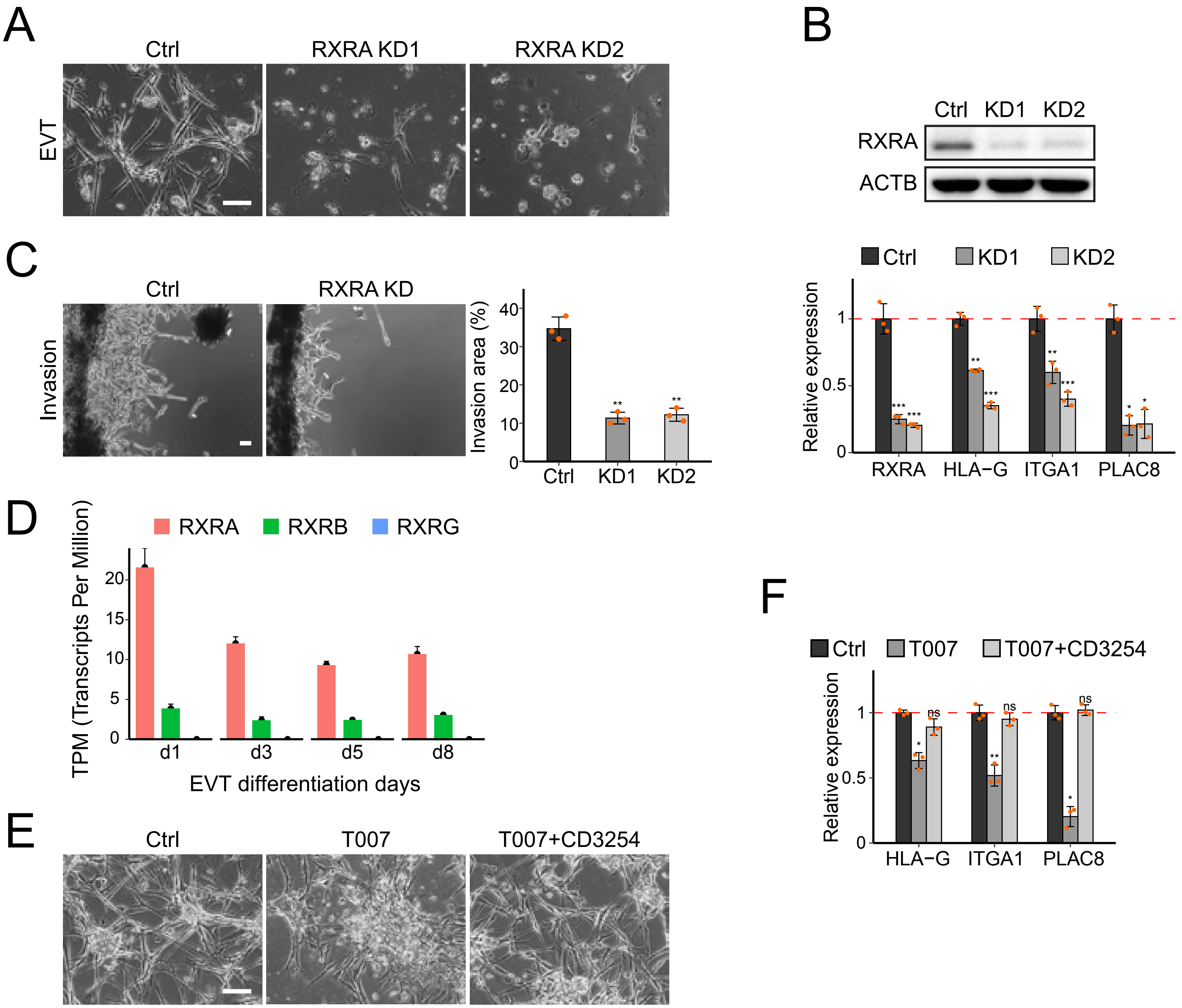

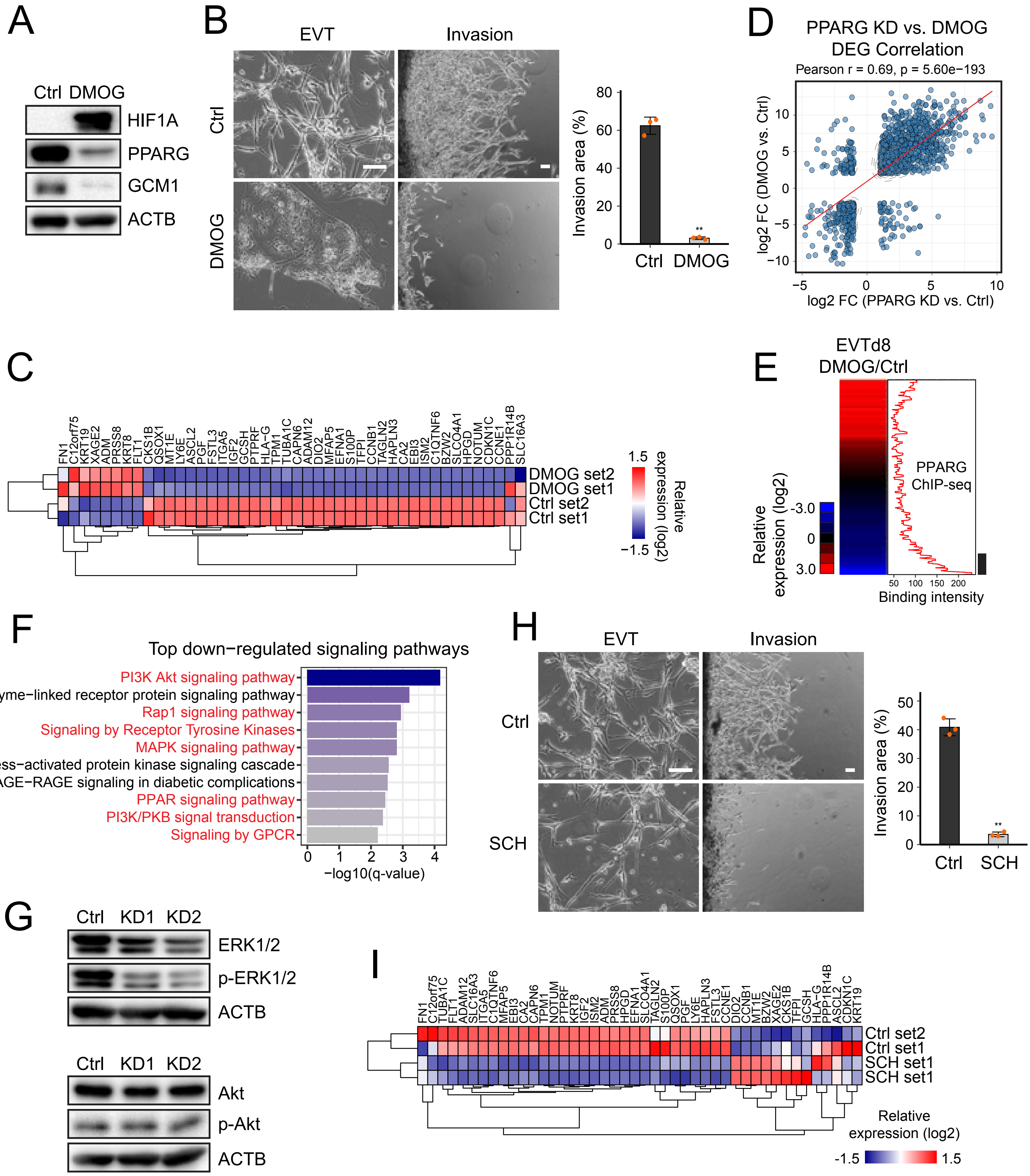
A
Ctrl DMOG
HIF1A
PPARG
GCM1
ACTB
B
EVT
Invasion
Ctrl
DMOG
Invasion area (%)
Ctrl DMOG
D
PPARG KD vs. DMOG
DEG Correlation
Pearson r = 0.69, p = 5.60e−193
log2 FC (DMOG vs. Ctrl)
log2 FC (PPARG KD vs. Ctrl)
C
DMOG set2
DMOG set1
Ctrl set2
Ctrl set1
Relative expression (log2)
E
EVTd8
DMOG/Ctrl
PPARG ChIP-seq
Relative expression (log2)
Binding intensity
F
Top down−regulated signaling pathways
PI3K Akt signaling pathway
Enzyme−linked receptor protein signaling pathway
Rap1 signaling pathway
Signaling by Receptor Tyrosine Kinases
MAPK signaling pathway
Stress−activated protein kinase signaling cascade
AGE−RAGE signaling in diabetic complications
PPAR signaling pathway
PI3K/PKB signal transduction
Signaling by GPCR
−log10(q-value)
H
EVT
Invasion
Ctrl
SCH
Invasion area (%)
Ctrl SCH
G
Ctrl KD1 KD2
ERK1/2
p-ERK1/2
ACTB
Ctrl KD1 KD2
Akt
p-Akt
ACTB
I
Ctrl set2
Ctrl set1
SCH set1
SCH set1
Relative expression (log2)
-1.5 1.5

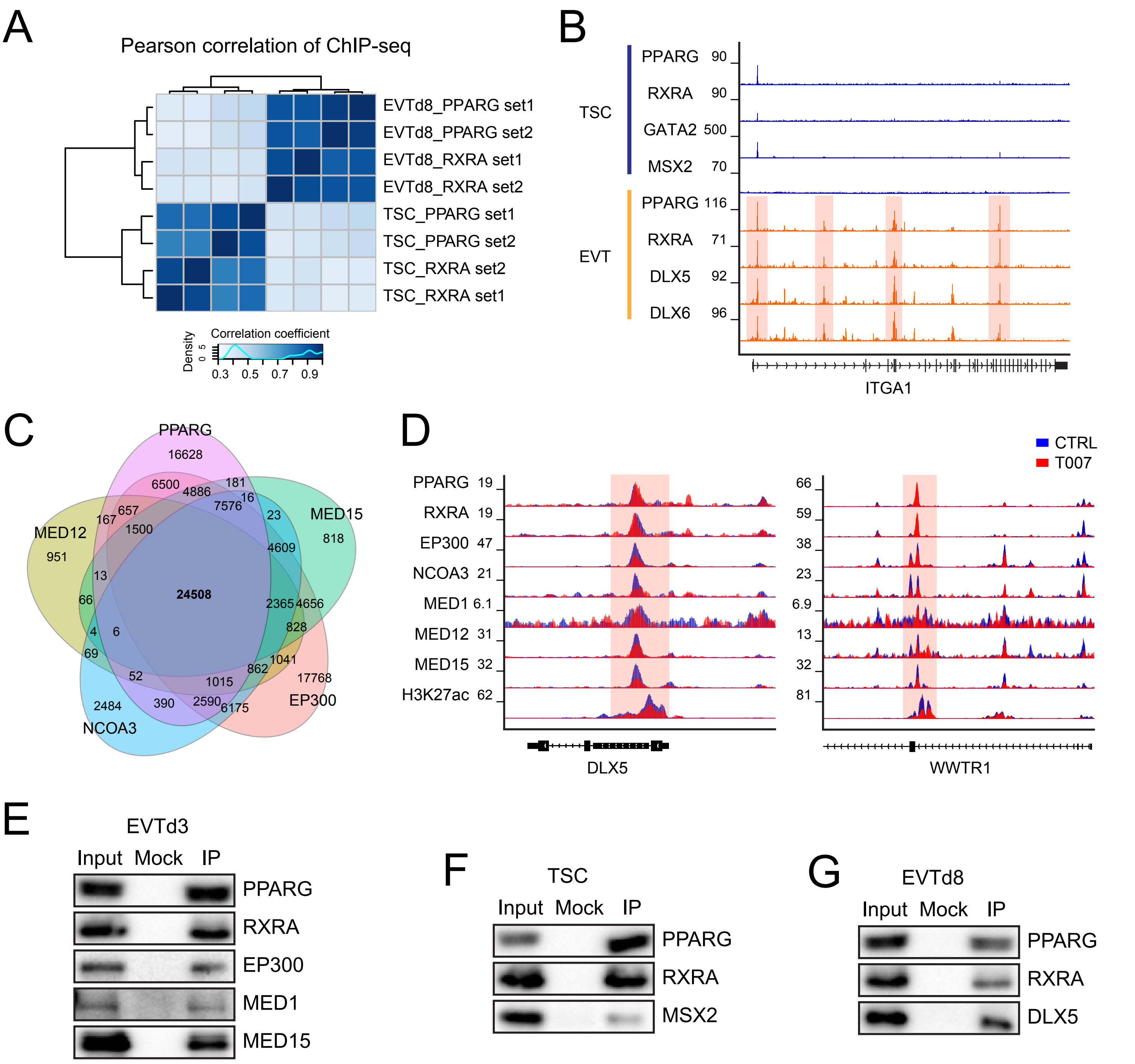
A
Pearson correlation of ChIP-seq
EVTd8_PPARG set1
EVTd8_PPARG set2
EVTd8_RXRA set1
EVTd8_RXRA set2
TSC_PPARG set1
TSC_PPARG set2
TSC_RXRA set2
TSC_RXRA set1
Correlation coefficient
Density
0 5
0.3 0.5 0.7 0.9
B
TSC
PPARG 90
RXRA 90
GATA2 500
MSX2 70
EVT
PPARG 116
RXRA 71
DLX5 92
DLX6 96
ITGA1
C
PPARG
16628
MED15
818
MED12
951
NCOA3
2484
EP300
17768
24508
6500
4886
181
16
7576
23
167
657
1500
4609
13
66
2365
4656
4
6
828
69
1041
862
52
1015
390
2590
6175
D
CTRL
T007
PPARG 19
RXRA 19
EP300 47
NCOA3 21
MED1 6.1
MED12 31
MED15 32
H3K27ac 62
66
59
38
23
6.9
13
32
81
DLX5
WWTR1
E
EVTd3
Input Mock IP
PPARG
RXRA
EP300
MED1
MED15
F
TSC
Input Mock IP
PPARG
RXRA
MSX2
G
EVTd8
Input Mock IP
PPARG
RXRA
DLX5
